# Disconcordance in Statistical Models of Bisphenol A and Chronic Disease Outcomes in NHANES 2003-08

**DOI:** 10.1371/journal.pone.0079944

**Published:** 2013-11-06

**Authors:** Martin F. Casey, Matthew Neidell

**Affiliations:** 1 Department of Health Policy and Management, Mailman School of Public Health, Columbia University, New York, New York, United States of America; 2 Icahn School of Medicine at Mount Sinai, New York, New York, United States of America; University of California, Berkeley, United States of America

## Abstract

**Background:**

Bisphenol A (BPA), a high production chemical commonly found in plastics, has drawn great attention from researchers due to the substance’s potential toxicity. Using data from three National Health and Nutrition Examination Survey (NHANES) cycles, we explored the consistency and robustness of BPA’s reported effects on coronary heart disease and diabetes.

**Methods And Findings:**

We report the use of three different statistical models in the analysis of BPA: (1) logistic regression, (2) log-linear regression, and (3) dose-response logistic regression. In each variation, confounders were added in six blocks to account for demographics, urinary creatinine, source of BPA exposure, healthy behaviours, and phthalate exposure. Results were sensitive to the variations in functional form of our statistical models, but no single model yielded consistent results across NHANES cycles. Reported ORs were also found to be sensitive to inclusion/exclusion criteria. Further, observed effects, which were most pronounced in NHANES 2003-04, could not be explained away by confounding.

**Conclusions:**

Limitations in the NHANES data and a poor understanding of the mode of action of BPA have made it difficult to develop informative statistical models. Given the sensitivity of effect estimates to functional form, researchers should report results using multiple specifications with different assumptions about BPA measurement, thus allowing for the identification of potential discrepancies in the data.

## Introduction

Bisphenol A (BPA) is an organic compound, commonly used in the synthesis of plastics and epoxy resins [1]. It is estimated that roughly ~2.2 million tons of BPA were produced in 2010, making it one of the world’s mostly widely manufactured chemicals [2]. Due to its hormone-like properties, BPA has attracted significant attention as a potentially toxic substance. To date over 5000 papers have been published to assess exposure to and toxicity of BPA [3]. Yet there remains little consensus whether BPA represents a true threat to consumer health [1,4], greatly complicating matters for the general public and policy-makers. 

Most experts agree that there is widespread exposure to BPA. The Centers for Disease Control and Prevention (CDC) reported detectable levels of BPA in 93% of Americans in 2003-04 [5]. Similarly high levels of BPA exposure have also been reported in Canadian, European, and Asian populations [6,7]. It is believed that BPA most commonly enters the body via the digestive tract. Studies have shown that BPA can leach out of food packaging and bottling materials, leading to widespread ingestion of the chemical [8,9]. Less common dermal, via cash register receipts, and airborne, via dust inhalation, exposures have also been observed [1,10]. 

Unlike exposure assessments, the toxicity of BPA remains hotly debated. As a weak endocrine disruptor, BPA has the potential to alter hormonally regulated processes in the human body [11]. Latent and sustained exposure to BPA has been linked to a battery of chronic conditions including: cancer, namely of the prostate [12,13], cardiac arrhythmias [14], disrupted pancreatic β-cell function, which increases risk for type II diabetes [15],, reduced fertility [16], asthma [17], metabolic syndrome [18], and obesity [19]. BPA, also believed to demonstrate teratogenic effects, may alter neurocognitive development in exposed embryos and fetuses [13,20]. However, these toxicology studies have failed to gain wide spread acceptance for numerous reasons including: difficulty extrapolating data from animal models to humans, lack of consistency in study end points, conflicting reports, and uncertainties in exposure assessment [3]. 

Epidemiological examinations of BPA toxicity have yielded similarly mixed results. A 2008 study, by Lang et al., reported statistically significant relationships between BPA exposure and cardiovascular disease (CVD) and diabetes in the National Health and Nutrition Examination Survey (NHANES) [21]. However, subsequent efforts to produce comparable results have yielded mixed results, with some supporting [22,23] and others challenging [24-26]. We hypothesized that conflicting epidemiological results could be attributed to one of two reasons: (1) positive associations could be explained away by confounding, or (2) results were sensitive to the functional form of the applied statistical model. In order to test our hypotheses, we replicated the results from Lang et al.’s paper and extended their work by including multiple NHANES cycles to increase sample size, expanding the number of covariates included in the regression models, and varying the functional form of the statistical model. 

## Materials and Methods

NHANES is a biennial, cross-sectional survey that measures the health status and dietary patterns of the non-institutionalized, civilian US population [27]. The survey uses a stratified, multistage probability design to identify a representative subset of the US population. NHANES subjects undergo several days of questionnaires and tests, yielding a wide range of information on demographics, socioeconomics, nutrition, medical status, dental status, and physiological/laboratory measurements. This study includes data from three NHANES cycles: 2003-04, 2005-06, and 2007-08, which will now be referred to as 03-04, 05-06, and 07-08 respectively. 

### Ethics Statement

With approval from the National Center for Health Statistics IRB, the Centers for Disease Control and Prevention (CDC) releases NHANES data to the public. All participants in the survey are required to provide informed consent. 

### Exposure and Outcome Assessment

During each NHANES cycle, one third of the subjects were randomly selected to provide urine samples. Urinary BPA, free and conjugated, was then measured by high-performance liquid chromatography-isotope dilution tandem mass spectroscopy (MS/MS) with peak focusing [28]. All measurements were completed by the Division of Environmental Health Laboratory Sciences at the National Center for Environmental Health. All measurements below the lower limit of detection (LLOD) - measured at 0.36 ng/ml in 03-04 and 0.40 ng/ml in 05-06 and 07-08 - are known to be unreliable. Consistent with prior studies, we assigned a value of LLOD/√2, or 0.28 ng/ml, to all values below the limit in each NHANES cycle [23,24]. 

Health outcomes, both coronary heart disease (CHD) and diabetes, were self-report measures collected via questionnaire. Subjects were asked if a doctor or other health professional had ever told them that he/she has CHD or diabetes. For the purposes of our analysis, subjects reporting borderline diabetes (defined as having blood sugars higher than normal, but not high enough to be classified as diabetes) were considered disease positive. To address the problem of multiple comparisons, when drawing conclusions over two outcomes (CHD and diabetes), we took the conservative Bonferoni approach and treated p<0.025 as being indicative of statistical significance.

### Study Population

Subjects ranged in age from 18-74. Children were excluded as the diseases of interest are rarely observed in their age group. The elderly, >74 years, were excluded to minimize the occurrence of co-morbidities in the study population. One subject was excluded from the 03-04 for missing a urinary creatinine value, while another subject was omitted from 05-06 for having an implausible mono-isobutyl phthalate concentration (nearly ~2 orders of magnitude larger than the next biggest value). Prior studies also excluded subjects for having urinary concentrations in excess of >80.1 ng/ml [23,24]. However, the aforementioned exclusion rule was based on the range of BPA values observed in the 03-04 dataset, rather than biologic plausibility [23]. Statistical models initially included subjects in the full range of reported BPA concentrations in each NHANES cycle. In order to assess sensitivity to BPA-based exclusion criteria, we repeated our analysis two times: once omitting subjects with BPA concentration >80.1 ng/ml, and again by omitting subjects below the LLOD and above the 99^th^ percentile. The first exclusion called for the removal of 9 subjects, while the second dropped another 404 subjects. 

### Statistical Analysis

NHANES uses a complex, multistage, probability sampling design to ensure generalizability of the results to the larger US population. In accord with NHANES Analytic and Reporting Guidelines, weighted estimates were calculated, including multi-year sampling weights [29]. Confidence intervals were calculated, with the provided ‘masked variance pseudo-psu’ and ‘pseudo-stratum’ variables, using a Taylor Series Linearization method. Analysis was carried out in Stata /IC v.12.1. 

Throughout the analysis, the parameter of interest is the relationship between BPA and either CHD or diabetes. Ideally, one would like to know the causal relationship between BPA and each outcome, but this is unlikely to be achieved using observational data without any (quasi-) random assignment. As one way of gauging whether we have in fact estimated a causal parameter is to assess how robust estimates are to alternative model specifications.

We initially replicated the results from Lang et al.’s study on the 2003-04 NHANES data [21]. Using logistic regressions, the authors created two models to estimate odd ratios (ORs) of self-reported disease per 1-standard deviation increase in BPA concentration. The first model adjusted for age, sex, and urinary creatinine concentration (**Model 1**). The second model expanded the regression model to include race/ethnicity, income, smoking, body mass index, and waist circumference (**Model 2**). We created four more models to probe the importance of potential confounders. Each model involved sequentially adding a new block of covariates. First, we adjusted for additional demographic information including: veteran/military status, citizenship status, marital status, household size, pregnancy status, language at subject interview, health insurance coverage, and employment status in the prior week (**Model 3**). We further controlled for potential sources of BPA exposure including: consumption of bottled water in the past 24 hrs [30], consumption of alcohol [9], and annual consumption of tuna fish [31] (**Model 4**). We then adjusted for ‘healthy behaviours’ as exhibited by: the presence of emotional support in one’s life, being on a diet, using a water treatment device, access to a routine source of health care, being vaccinated for Hepatitis A or B, consumption of dietary supplements (vitamins or minerals), and inability to purchase balanced meals on a consistent basis (**Model 5**). Finally, we controlled for exposure to phthalates, another potentially toxic substance commonly found in plastics with BPA [32]. Our regression model includes three metabolites commonly found in the human body after phthalate consumption: (2-ethylhexyl) phthalate (MEHP), mono-isobutyl phthalate (MiBP), and mono-n-butyl phthalate (MeBP) (**Model 6**). All pooled analyses controlled for survey cycle as well. Phthalate data was not available in 03-04; thus odds ratios (ORs), for Model 6, are not reported for the 03-04 survey, nor the pooled data. Tables S1-S4 provide further information on covariates, such as their unadjusted correlations to BPA and NHANES variable identifier, included in Models 3-6. Each model was compared to the prior model, in the pooled data, using the Hausman statistic [33], Akaike information criterion (AIC) and Bayesian information criterion (BIC). 

Urinary creatinine was included in all regression models to adjust for variable diluteness in spot urine samples [34]. Creatinine was measured using a Jaffe reaction in 03-04 and 05-06 and using an enzymatic method in 07-08. Creatinine measurements were made with Beckman CX3 Analyzer and Roche/Hitachi ModP Analyzer in 03-04/05-06 and 07-08 respectively. We used NHANES recommended stepwise transformation on the 03-04/05-06 data to account for interference effects of the Jaffe reaction, making the creatinine data between NHANES cycles more comparable [35]. 

Finally, we varied the functional form of our statistical model by mimicking two alternatives found in literature: a log-linear exposure-response relationship [24] and a dose-response logistic regression [22]. In the first variation, we carried out logistic regression analysis with a log-transformed urinary BPA values, thus creating a log-linear relationship between exposure and response. The log-linear model can better account for diminishing linearity, as outcomes plateau, in high-dose exposure regions [36]. For consistency across body metabolites, phthalate and creatinine measurements were log-transformed in this model as well. In the dose-response regression, exposure was transformed into a categorical variable by quartile of BPA exposure (<1.1 ng/ml, 1.2-2.2 ng/ml, 2.3-4.2 ng/ml, and >4.2 ng/ml), with the lowest quartile serving as the reference group. If the assumptions of linearity hold, then we would expect to see steadily increasing ORs across increasing quartiles of exposure. The dose-response analysis may help uncover whether adverse effects, if observed, plateau in high-dose regions. Analysis of marginal effects was carried out, in each functional form variation, to assess how diabetes and CHD risk changed with small changes in BPA exposure. Finally, analyses were repeated with an interaction term between BPA and survey cycle, in order to assess the consistency of estimates across cycles. 

## Results

**Figure 1:**
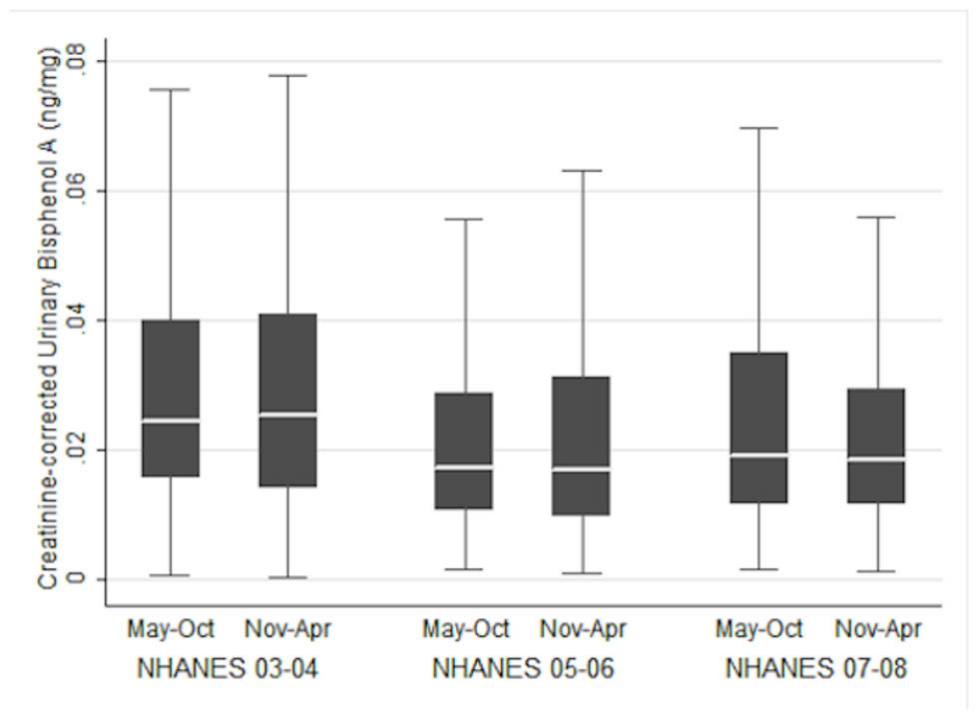
A box-and-whisker plot of creatinine-corrected and weighted Bisphenol A exposure in NHANES 03-04 (N = 1,455), 05-06 (N = 1,498), 07-08 (N = 1,705). Outliers are not depicted.

The entire sample included 4658 subjects with 1455 in 03-04, 1498 in 05-06, and 1705 in 07-08. Unadjusted mean BPA concentrations dropped across survey cycles from 4.78 to 4.16 to 3.76 ng/ml in 03-04, 05-06, and 07-08 respectively. Median BPA exposure followed a similar, but less dramatic, downward trend across NHANES cycles, as it decreased from 2.8 to 2.1 to 2 ng/ml. Figure 1 provides a box plot with median, 25^th^ percentile, and 75^th^ percentile creatinine-corrected BPA concentrations across NHANES survey cycles. Furthermore, only 4,211 subjects reported their CHD status, with 144 responding in the affirmative. Similarly, 487 out of 4,654 subjects reported having diabetes or borderline diabetes. 

Table 1 provides a breakdown, with weighted percentages, of demographic characteristics of the subjects in each NHANES cycles. In each survey cycle, subjects were separated by low and high exposure, based off their urinary BPA concentration relative to the median value. Roughly 52% of individuals with high exposure were male, while only ~46% of those with low exposure were male, suggesting an increased risk for BPA exposure among males. Table 1 further suggests that younger subjects, non-Hispanic blacks, individuals with a HS degree or equivalent, families in lower income brackets, individuals with a high BMI, and current smokers are at risk for increased BPA exposure. Tables S1-S4 provide further information on the characteristics, sources of BPA exposure, and patterns of healthy behaviour in the participants across NHANES cycles. 

**Table 1:** Weighted percentages of sociodemographic characteristics separated by NHANES cycle and level of exposure, where Low ≤2.2 ng/ml and High >2.2 ng/ml.

		NHANES 03-04		NHANES 05-06		NHANES 07-08		Pooled		p-value^a^
		N = 1455		N = 1498		N = 1705		N = 4658		
		Low (%)	High (%)	Low (%)	High (%)	Low (%)	High (%)	Low (%)	High (%)	
Sex	Male	43.63	51.76	45.34	53.9	48.76	49.99	46.08	51.81	<0.001
	Female	56.37	48.24	54.66	46.1	51.24	50.01	53.92	48.19	
Age Group	18-29	17.45	28.13	16.94	31.1	18.54	30.26	17.64	29.7	<0.001
	30-39	17.63	22.45	19.92	19.54	18.88	19.77	18.93	20.72	
	40-49	24.33	21.69	22.68	21.74	22.86	20	23.19	21.15	
	50-59	19.08	16.6	21.73	14.28	20.55	17.61	20.59	16.25	
	60-74	21.52	11.13	18.73	13.35	19.18	12.36	19.65	12.18	
Race/Ethnicity	Mexican American	8.58	8.36	7.82	8.96	9.36	8.85	8.57	8.7	<0.001
	Other Hispanic	4.49	4.13	2.98	4.02	4.81	4.95	4.03	4.37	
	Non-Hispanic white	70.53	68.24	74.07	65.65	69.64	67.51	71.55	67.24	
	Non-Hispanic black	6.95	15.18	9.52	15.97	8.69	15.38	8.52	15.48	
	Other	9.46	4.09	5.61	5.4	7.5	3.31	7.33	4.22	
Education	Less than <HS	17.5	18.5	13.91	19.06	19.5	19.3	16.86	18.92	0.010
	HS or equivalent	23.24	27.87	23.6	26.35	24.87	27.43	23.95	27.28	
	Greater than HS	59.25	53.64	62.49	54.34	55.62	53.27	59.19	53.72	
	Unknown	0	0	0	0.26	0	0	0	0.07	
Household	<$20,000	13.61	17.72	10.45	16.54	12.71	13.29	12.11	15.93	<0.001
Income	$20,000-$35,000	17.07	18.97	14.59	18.77	15.22	17.62	15.49	18.47	
	$35,000-$65,000	25.96	27.74	25.94	26.28	21.2	24.76	24.28	26.34	
	>$65,000	37.22	30.41	44.32	34.36	45.36	37.93	42.75	34.03	
	Unknown	6.14	5.17	4.71	4.05	5.51	6.4	5.38	5.24	
BMI	<18.5	2.03	2.22	3.17	2.01	0.99	1.49	2.09	1.92	0.005
	18.5-24.9	36.21	31.66	32.49	28.34	35.8	30.22	34.67	30.22	
	25.0-29.9	33.09	28.32	32.03	30.87	30.33	29.58	31.72	29.48	
	30-34.9	17.94	21.55	16.9	21.16	19.21	17.81	18	20.21	
	>=35	9.13	14.6	14.65	17.18	12.36	20.47	12.34	17.27	
	Unknown	1.59	1.66	0.76	0.45	1.31	0.42	1.18	0.9	
Smoking Status	Never smoked	51.58	46.56	50.01	46.98	48.87	50.89	50.04	48.1	0.005
	Former smoker	24.91	20.79	23.25	19.95	24.09	22	24	20.94	
	Some days	3.73	4.93	3.33	2.92	4.26	4.11	3.77	4.07	
	Every day	17.86	22.56	21.33	24.37	19.11	17.83	19.6	21.54	
	Unknown	1.91	5.17	2.08	5.78	3.67	5.18	2.59	5.35	

a **Pearson’s chi-square tests were carried out, using survey weights, on the data from the pooled sample**.

Odds ratios (ORs) from the sequential logistic regression models, both linear and log-linear, can be found in Tables 2-4, as well as Tables S5-S12. Table 2 provides ORs for associating CHD with a 1-standard deviation (1-SD) increase in BPA, separately by NHANES cycle and all cycles pooled together. The ORs for 03-04 were statistically significant. The ORs for 05-06 were also statistically significant, but considerably smaller in magnitude. The ORs for 07-08 were even smaller and were no longer significant. The estimates from the pooled sample lie in between and were statistically significant. By contrast, Table 3 shows that ORs for diabetes indicate significantly increased risk in the 03-04 sample only. ORs in 05-06 are not statistically significant, while ORs in 07-08 demonstrated a non-significant protective effect. ORs remained fairly stable, for both CHD and diabetes, with the addition of covariates from Model 1 to Model 6. Further, the Hausman test never reached statistical significance, confirming that confounding variables had very little impact on effect estimates. However, the overall fit of the model always improved as covariates were added as indicated by decreasing trends in both AIC and BIC over the various model specifications (Tables S13-S14). 

**Table 2:** Logistic regression analysis of self-reported CHD per standard deviation increase of Bisphenol A exposure for NHANES 03-04 (N = 1,455), 05-06 (N = 1,498), 07-08 (N = 1,705), and a pooled sample (N = 4,658).

	NHANES 03-04		NHANES 05-06		NHANES 07-08		Pooled		
	OR (95% CI)		OR (95% CI)		OR (95% CI)		OR (95% CI)		Hausman Statistic (p) †
Model 1	1.408*	(1.093 - 1.815)	1.130	(0.959 - 1.331)	1.058	(0.888 - 1.259)	1.140*	(1.025 - 1.268)	--
Model 2	1.596*	(1.126 - 2.262)	1.179	(1.014 - 1.371)	1.116	(0.921 - 1.353)	1.163*	(1.038 - 1.303)	0.998 (0.607)
Model 3	1.713**	(1.180 - 2.486)	1.243	(1.020 - 1.516)	1.101	(0.883 - 1.372)	1.158*	(1.026 - 1.308)	0.05 (0.975)
Model 4	1.855**	(1.248 - 2.758)	1.293*	(1.058 - 1.580)	1.123	(0.890 - 1.418)	1.147*	(1.023 - 1.287)	<0 (1)
Model 5	1.824**	(1.288 - 2.583)	1.267*	(1.041 - 1.542)	1.123	(0.854 - 1.476)	1.136	(1.014 - 1.273)	<0 (1)
Model 6	--	--	1.284*	(1.047 - 1.574)	1.131	(0.860 - 1.487)	--	--	--

* p < 0.025 ; ** p < 0.01

Model 1: adjusted for age, sex, and urinary creatinine concentration

Model 2: further adjusted for race/ethnicity, income, smoking, body mass index, and waist circumference

Model 3: veteran/military status, citizenship status, marital status, household size, pregnancy status, language at subject interview, health insurance coverage, and employment status in the prior week

Model 4: consumption of bottled water in the past 24 hrs, consumption of alcohol, and annual consumption of tuna fish

Model 5: presence of emotional support in one’s life, being on a diet, using a water treatment device, access to a routine source of health care, vaccinated for Hepatitis A or B, consumption of dietary supplements (vitamins or minerals), and inability to purchase balanced meals on a consistent basis

Model 6: concentration of (2-ethylhexyl) phthalate (MEHP), mono-isobutyl phthalate (MiBP), and mono-n-butyl phthalate (MeBP)

† Hausman test was performed to compare the fit of each model to the prior model in the pooled data

**Table 3:** Logistic regression analysis of self-reported diabetes per standard deviation increase of Bisphenol A exposure for NHANES 03-04 (N = 1,455), 05-06 (N = 1,498), 07-08 (N = 1,705), and a pooled sample (N = 4,658).

	NHANES 03-04		NHANES 05-06		NHANES 07-08		Pooled		
	OR (95% CI)		OR (95% CI)		OR (95% CI)		OR (95% CI)		Hausman Statistic (p) †
Model 1	1.404**	(1.210 - 1.628)	0.984	(0.805 - 1.203)	0.722	(0.526 - 0.991)	1.053	(0.958 - 1.157)	--
Model 2	1.400**	(1.245 - 1.574)	0.975	(0.723 - 1.316)	0.710	(0.506 - 0.995)	1.074	(0.980 - 1.176)	<0 (1)
Model 3	1.358**	(1.237 - 1.491)	1.032	(0.847 - 1.258)	0.709	(0.496 - 1.015)	1.077	(0.986 - 1.176)	<0 (1)
Model 4	1.359**	(1.187 - 1.556)	1.037	(0.866 - 1.241)	0.711	(0.487 - 1.038)	1.072	(0.982 - 1.170)	<0 (1)
Model 5	1.398**	(1.183 - 1.653)	1.008	(0.861 - 1.181)	0.716	(0.500 - 1.025)	1.065	(0.973 - 1.166)	0.099 (0.952)
Model 6	--	--	1.007	(0.859 - 1.182)	0.699	(0.464 - 1.052)	--	--	--

* - p < 0.025 ; ** p < 0.01

Model 1: adjusted for age, sex, and urinary creatinine concentration

Model 2: further adjusted for race/ethnicity, income, smoking, body mass index, and waist circumference

Model 3: veteran/military status, citizenship status, marital status, household size, pregnancy status, language at subject interview, health insurance coverage, and employment status in the prior week

Model 4: consumption of bottled water in the past 24 hrs, consumption of alcohol, and annual consumption of tuna fish

Model 5: presence of emotional support in one’s life, being on a diet, using a water treatment device, access to a routine source of health care, vaccinated for Hepatitis A or B, consumption of dietary supplements (vitamins or minerals), and inability to purchase balanced meals on a consistent basis

Model 6: concentration of (2-ethylhexyl) phthalate (MEHP), mono-isobutyl phthalate (MiBP), and mono-n-butyl phthalate (MeBP)

† Hausman test was performed to compare the fit of each model to the prior model in the pooled data

**Table 4:** Log-linear analysis of self-reported CHD per ten-fold increase in Bisphenol A exposurefor NHANES 03-04 (N = 1,455), 05-06 (N = 1,498), 07-08 (N = 1,705), and a pooled sample (N = 4,658).

	NHANES 03-04		NHANES 05-06		NHANES 07-08		Pooled		
	OR (95% CI)		OR (95% CI)		OR (95% CI)		OR (95% CI)		Hausman Statistic (p) †
Model 1	1.527	(0.918 - 2.542)	1.126	(0.698 - 1.815)	1.416	(0.920 - 2.180)	1.320	(1.025 - 1.698)	--
Model 2	1.860	(1.023 - 3.383)	1.124	(0.741 - 1.706)	1.493	(0.994 - 2.242)	1.326	(1.035 - 1.698)	0.123 (0.941)
Model 3	1.815*	(1.155 - 2.852)	1.169	(0.790 - 1.730)	1.564	(1.039 - 2.356)	1.323*	(1.037 - 1.688)	0.005 (0.998)
Model 4	1.661**	(1.184 - 2.331)	1.233	(0.776 - 1.960)	1.616	(1.048 - 2.493)	1.302	(1.008 - 1.681)	0.271 (0.873)
Model 5	1.584	(1.066 - 2.354)	1.178	(0.765 - 1.815)	1.649	(1.025 - 2.654)	1.280	(0.993 - 1.649)	0.476 (0.788)
Model 6	--	--	1.460	(0.893 - 2.386)	1.679	(1.007 - 2.801)	--	--	--

* p < 0.025 ; ** p < 0.01

Model 1: adjusted for age, sex, and urinary creatinine concentration

Model 2: further adjusted for race/ethnicity, income, smoking, body mass index, and waist circumference

Model 3: veteran/military status, citizenship status, marital status, household size, pregnancy status, language at subject interview, health insurance coverage, and employment status in the prior week

Model 4: consumption of bottled water in the past 24 hrs, consumption of alcohol, and annual consumption of tuna fish

Model 5: presence of emotional support in one’s life, being on a diet, using a water treatment device, access to a routine source of health care, vaccinated for Hepatitis A or B, consumption of dietary supplements (vitamins or minerals), and inability to purchase balanced meals on a consistent basis

Model 6: concentration of (2-ethylhexyl) phthalate (MEHP), mono-isobutyl phthalate (MiBP), and mono-n-butyl phthalate (MeBP)

† Hausman test was performed to compare the fit of each model to the prior model in the pooled data

Most ORs from the log-linear model for CHD achieved significance at the 95% confidence level in 03-04, 07-08, and pooled sample (Table 4). However significance was lost in most instances after application of the Bonferoni correction. Applying the log-linear transformation to self-reported diabetes yielded similar results (in comparison to logistic regression analysis, Table 2), with the exception that statistical significance was achieved in the pooled sample (Table 5). Similar to the linear model, ORs were robust to confounding as indicated by non-significant Hausman tests. In terms of marginal effects, a one standard deviation increase in BPA was associated with a 0.3 to 0.5 increase in the probability of CHD and diabetes across the various models, while ten-fold increases in BPA were associated with 0.6 to 1.3 percent changes (Tables S17-S20). Marginal effects, for the logistic model, were significant for CHD, but not for diabetes. The opposite pattern of significance was observed in the log-linear model. Given that the rate of CHD and diabetes were relatively small in the study population (3.5 and 11.7% respectively), these changes in risk were rather substantial. 

**Table 5:** Log-linear analysis of self-reported diabetes per ten-fold increase in Bisphenol A exposure for NHANES 03-04 (N = 1,455), 05-06 (N = 1,498), 07-08 (N = 1,705), and a pooled sample (N = 4,658).

	NHANES 03-04		NHANES 05-06		NHANES 07-08		Pooled		
	OR (95% CI)		OR (95% CI)		OR (95% CI)		OR (95% CI)		Hausman Statistic (p) †
Model 1	1.562**	(1.323 - 1.844)	1.203	(0.947 - 1.529)	0.961	(0.790 - 1.170)	1.209**	(1.071 - 1.365)	--
Model 2	1.548**	(1.315 - 1.823)	1.171	(0.838 - 1.636)	0.967	(0.812 - 1.151)	1.197*	(1.039 - 1.380)	(0.146)
Model 3	1.524**	(1.295 - 1.794)	1.223	(0.883 - 1.695)	0.959	(0.792 - 1.163)	1.205*	(1.048 - 1.386)	(<0)
Model 4	1.476**	(1.286 - 1.694)	1.250	(0.879 - 1.777)	0.934	(0.756 - 1.153)	1.198*	(1.040 - 1.380)	(0.446)
Model 5	1.492**	(1.267 - 1.757)	1.230	(0.894 - 1.694)	0.932	(0.759 - 1.146)	1.202**	(1.049 - 1.377)	(0.026)
Model 6	--	--	1.214	(0.881 - 1.672)	0.870	(0.679 - 1.115)	--	--	--

* p < 0.025 ; ** p < 0.01

Model 1: adjusted for age, sex, and urinary creatinine concentration

Model 2: further adjusted for race/ethnicity, income, smoking, body mass index, and waist circumference

Model 3: veteran/military status, citizenship status, marital status, household size, pregnancy status, language at subject interview, health insurance coverage, and employment status in the prior week

Model 4: consumption of bottled water in the past 24 hrs, consumption of alcohol, and annual consumption of tuna fish

Model 5: presence of emotional support in one’s life, being on a diet, using a water treatment device, access to a routine source of health care, vaccinated for Hepatitis A or B, consumption of dietary supplements (vitamins or minerals), and inability to purchase balanced meals on a consistent basis

Model 6: concentration of (2-ethylhexyl) phthalate (MEHP), mono-isobutyl phthalate (MiBP), and mono-n-butyl phthalate (MeBP)

† Hausman test was performed to compare the fit of each model to the prior model in the pooled data

In the analysis of CHD risk, application of initial exclusion criteria, or removing subjects with BPA >80.1 ng/ml, led to a near ~30% increase in estimated ORs in the pooled data (Table S2 and S5). Furthermore, excluding subjects below the LLOD and in the top percentile led to another large increase, nearly ~20%, in the estimated ORs from the pooled data (Tables S5 and S6). Comparable changes, often similar in direction but smaller in magnitude, were observed in log-linear and diabetes-related regression models with the application of exclusion criteria. Statistical significant ORs for diabetes, by the linear model, were only observed after exclusion of subjects >80.1 ng/ml in the pooled sample. Better model fits, or lower AIC and BIC, were observed after applying exclusion criteria (Table S13 and S14).

Finally, Table 5 provides estimated ORs from the dose-response regression analysis in the pooled sample. Although significant ORs were not observed, an upward trend in CHD risk was observed across BPA quartiles. Individuals in the 25-50^th^ percentile of BPA exposure, 1.1-2.2 ng/ml, did not appear to be at increased risk for CHD with respect to the referent group. Unlike CHD, significance was achieved in the majority of ORs for diabetes, but there was no dose-response relationship, with the middle upper quartile showing reduced risk with respect to the middle lower quartile. Expanded dose-response analysis for each NHANES cycles are reported in Tables S15 and S16. Significant interactions between BPA and NHANEs cycle were observed in the linear model, but not in the log-linear nor dose-response models (Table S22-S23).

## Discussion

The potential toxicity of BPA has become a topic of much debate among academics and policy-makers. The controversy expanded beyond the teratogenic effects of BPA when Lang et al reported statistically significant associations between BPA and self-reported CHD and diabetes in the 03-04 NHANES cycle [21]. Several subsequent studies have explored the issue further by altering the statistical model and/or expanding the data set to include more NHANES data [22-24,26]. Our study is meant to replicate the work of prior studies, expand the analysis to three NHANES cycles (03-04, 05-06, and 07-08), and to explore the issues of potential confounding and functional form. 

Our first major finding was that the ORs were generally robust to sources of potential confounding, either slightly increasing or remaining stable as we added more covariates to each model. We expanded upon previous studies to include four categories of potential confounders: additional demographic information, differential exposure to potential BPA sources, likelihood to engage in healthy behaviours, and concurrent phthalate exposure. From the pooled data from Table 2 and Table 3, we see at most a 5% change in the estimated ORs with each expansion of the regression model. Further, all Hausman tests failed to achieve significance, confirming that no one block of covariates significantly affected the estimation of associations between BPA and CHD/diabetes risk. Comparable results were observed across individual NHANES cycles and log-linear regression models. The stability across covariate models confirms that the estimated ORs were robust. However, our study was limited to assessment of confounders that were consistently reported in NHANES across three survey cycles. For example, additional sources of exposure may have also affected the health outcomes, such as number of meals away from home [9], use of hemodialysis [37], use of dental sealants [38], use of microwave containers [39], and more. Moreover, CHD medications, namely diuretics, may act to increase a subject’s BPA clearance rate, potentially creating a spurious relationship between BPA and CHD [24]. 

We also examined whether consistency was achieved across NHANES cycles and the pooled data, and found ORs to vary considerably across cycles. Figure 1 demonstrates that BPA exposure was highest in the 03-04 cycle, and relatively similar from 05-06 to 07-08. Mean BPA concentration decreased from 4.76 (SD = 6.78) to 4.16 (SD = 13.28) to 3.77 ng/ml (SD = 7.00) in 03-04, 05-06, and 07-08 respectively. It is unlikely that decreases in BPA exposure from 03-04 to 05-06 can be attributed to increased public awareness/concern of BPA. Major media outlets - including the New York Times, NPR, and NBC News – did not start reporting the potential harms of BPA until after the first half of the 05-06 NHANES cycle [40-42]. Silver et al. have suggested that the unusually high BPA concentrations in 03-04 may be attributed to a sampling error [24]. The authors used this argument to explain the unusual difference in effect estimates between the 03-04 data and other NHANES cycles (Table 3) in the assessment of diabetes. Although this result was replicated in our study, the pattern did not carry over to the analysis of CHD. 

Further complicating the matter is lack of consistency across linear and log-linear models in the assessment of CHD risk (Tables 2 and 4). The log-linear model failed to achieve significance in the pooled sample, while the linear model did (note that 03-04 yielded significant OR estimates in both instances, while 05-06 and 07-08 samples were generally non-significant). A similar inconsistency was observed in the analysis of self-reported diabetes (Tables 3 and 5), where statistical significance was subsequently achieved in the pooled sample after application of the log-transformation. This result is representative of the second major finding of our study: observed ORs, were sensitive to the functional-form of the applied statistical model. This is disconcerting because medical theories offer little guidance on the appropriate choice of functional form. Several papers have advocated for researchers to use a log-linear model as it better estimates risk in samples with high-exposure regions [24,36]. AIC and BIC generally indicated that the log-linear model fit the data better (Table S13 and S14), but changes in criterion were very small across functional-forms. We advise researchers to report results from multiple specifications to identify any potential discrepancies while also allowing readers to judge for themselves. Further, researchers should be statistically rigorous when reporting across multiple outcomes as ORs were noted to lose significance after application of the Bonferoni correction. 

We also attempted to explore if there was a dose-response relationship between BPA and health and explore the influence of outliers. While we find a pattern of increasing ORs consistent for CHD, the estimates were not statistically significant. More dramatic results were observed for diabetic outcomes when a dose-response model was applied (Table 6): estimates were statistically significant, but ORs supported a highly nonlinear relationship. Furthermore, outliers also influenced results. Previous papers have omitted subjects with BPA greater than >80.1 ng/ml, but this criteria is insufficient as it is arbitrarily based off the range of values observed in the 03-04 NHANES dataset [23]. Exclusion of subjects with a urinary BPA concentration in excess of >80.1 ng/ml led to a ~30% and ~15% increase in the estimated CHD and diabetes ORs respectively. Even larger increases, ~60% and ~20%respectively, were observed as subjects below the LLOD and in the top percentile were excluded. The log-linear model was less sensitive to the effects of inclusion/exclusion criteria because the log-transformation tends to dampen the influence of outliers. While the use of dummy variables can alleviate the influence of outliers, our results for diabetes were inconsistent with a dose-response relationship. 

**Table 6:** Dose-response regression analysis of both CHD and diabetes in the pooled NHANES data (N = 4,658).

		CHD			Diabetes	
	[BPA] (ng/ml)	OR (95% CI)			OR (95% CI)	
Model 1	<1.1	Ref	--		Ref	--
	1.2-2.2	0.663	(0.346 - 1.272)		1.682**	(1.252 - 2.258)
	2.3-4.2	1.118	(0.606 - 2.066)		1.593	(1.036 - 2.451)
	>4.2	1.771	(0.875 - 3.583)		1.940**	(1.332 - 2.824)
Model 2	<1.1	Ref	--		Ref	--
	1.2-2.2	0.617	(0.331 - 1.149)		1.614**	(1.144 - 2.275)
	2.3-4.2	1.069	(0.575 - 1.987)		1.493	(0.947 - 2.355)
	>4.2	1.676	(0.861 - 3.262)		1.806**	(1.170 - 2.787)
Model 3	<1.1	Ref	--		Ref	--
	1.2-2.2	0.618	(0.332 - 1.150)		1.632**	(1.147 - 2.321)
	2.3-4.2	1.088	(0.584 - 2.025)		1.529	(0.989 - 2.363)
	>4.2	1.657	(0.865 - 3.177)		1.812**	(1.170 - 2.805)
Model 4	<1.1	Ref	--		Ref	--
	1.2-2.2	0.563	(0.291 - 1.092)		1.610*	(1.122 - 2.311)
	2.3-4.2	1.063	(0.533 - 2.116)		1.476	(0.958 - 2.276)
	>4.2	1.610	(0.810 - 3.200)		1.793*	(1.147 - 2.801)
Model 5	<1.1	Ref	--		Ref	--
	1.2-2.2	0.520	(0.250 - 1.084)		1.443	(0.982 - 2.119)
	2.3-4.2	1.006	(0.508 - 1.994)		1.512	(0.998 - 2.289)
	>4.2	1.520	(0.774 - 2.987)		1.760*	(1.137 - 2.724)

* p < 0.025 ; ** p < 0.01

Model 1: adjusted for age, sex, and urinary creatinine concentration

Model 2: further adjusted for race/ethnicity, income, smoking, body mass index, and waist circumference

Model 3: veteran/military status, citizenship status, marital status, household size, pregnancy status, language at subject interview, health insurance coverage, and employment status in the prior week

Model 4: consumption of bottled water in the past 24 hrs, consumption of alcohol, and annual consumption of tuna fish

Model 5: presence of emotional support in one’s life, being on a diet, using a water treatment device, access to a routine source of health care, vaccinated for Hepatitis A or B, consumption of dietary supplements (vitamins or minerals), and inability to purchase balanced meals on a consistent basis

Unfortunately, animal studies offer little clarity in the analysis of BPA. It has been argued that BPA may induce harmful biological changes as both an estrogen agonist and androgen antagonist [43,44]. Another study directly suggested that the estrogenic effects of BPA can induce insulin resistance in mice [45]. However, these highly suggestive results often remain isolated. In an extensive review, Hengstler et al. report that animal studies generally yielded inconsistent and opposing results, often pitting academic and industry researchers against one another [3]. Further complicating matters, BPA metabolism varies greatly across different species, making it difficult to extrapolate animal models to humans [46]. Significantly more research is required to substantiate any mechanistic pathway(s) that explain how BPA induces adverse effects in the human body. 

Significant interactions between BPA and NHANEs cycle were observed in the linear model, but not in the log-linear or dose-response models. We are unfortunately unable to test the reason behind these inconsistencies across years and models without additional information, but we suspect issues with the quality of BPA measurement is one important factor. Urinary output, and its chemical content, varies greatly over a 24hr period. Studies have suggested that single spot urinary samples are reasonable estimates for determining average population exposure to BPA, but only moderately sensitive in estimating an individual’s daily BPA intake [47,48]. Our analysis could better account for temporal variability in BPA concentration by adjusting for total 24hr urinary output [6,9], but this variable was not available in any NHANES cycle. Further, roughly 8% of the pooled sample (~366 subjects) was below the LLOD. This creates an unfortunate situation in which values below the LLOD are considered differently than values above the LLOD (where the former were uniformly assigned a value of LLOD/√2 and the latter measured on continuous spectrum). Without further information on the nature of error below the LLOD, we cannot rule out bias due to mismanagement of non-detects in our statistical models [49]. Finally, and potentially most importantly, single-spot urine assessment is a poor substitute for chronic exposure to BPA. In this regard, it is difficult to bridge the gap from cross-sectional studies to causal theories that link sustained insult, via BPA’s endocrine disruption, to the underlining pathologies of lifestyle diseases, such as CHD and diabetes. 

## Conclusions

The National Institute of Environmental Health Sciences recently allocated 30 million dollars to fund BPA research [50]. We hope that this study can inform researchers in their statistical modelling. While the results were generally robust to the sequential addition of more covariates, the ORs changed dramatically, sometimes even flipping signs, across statistical models. Unfortunately we could not pin down the reason for these changes. Therefore, our recommendation is that it is essential to report results from multiple specifications with different assumptions about BPA measurement and its potential health effects in order for these observational analyses to be informative on the health effects of BPA. 

## Supporting Information

Table S1 **Sociodemographic variables included in Model 3 and their unadjusted correlations with BPA.**
(DOCX)

Table S2 **Model 4 variables, sources of BPA exposure, and unadjusted correlations with BPA.**
(DOCX)

Table S3 **Model 5 variables, healthy behaviours, and unadjusted correlations with BPA.**
(DOCX)

Table S4 **Biomarker, creatinine and phthalate, and unadjusted correlations with BPA.**
(DOCX)

Table S5 **Logistic regression analysis of self-reported CHD, *excluding* subjects with [BPA] > 80.1 ng/ml, per standard deviation increase of Bisphenol A exposure for NHANES 03-04 (N = 1,455), 05-06 (N = 1,498), 07-08 (N = 1,705), and a pooled sample (N = 4,658).**
(DOCX)

Table S6 **Logistic regression analysis of self-reported CHD, *excluding* subjects [BPA]<LLOD and >99^th^ percentile, per standard deviation increase of Bisphenol A exposure for NHANES 03-04 (N = 1,455), 05-06 (N = 1,498), 07-08 (N = 1,705), and a pooled sample (N = 4,658).**
(DOCX)

Table S7 **Log-linear analysis of self-reported CHD, *excluding* subjects with [BPA] > 80.1 ng/ml, per ten-fold increase in Bisphenol A exposure for NHANES 03-04 (N = 1,455), 05-06 (N = 1,498), 07-08 (N = 1,705), and a pooled sample (N = 4,658).**
(DOCX)

Table S8 **Log-linear analysis of self-reported CHD, *excluding* subjects [BPA]<LLOD and >99^th^ percentile, per ten-fold increase in Bisphenol A exposure for NHANES 03-04 (N = 1,455), 05-06 (N = 1,498), 07-08 (N = 1,705), and a pooled sample (N = 4,658).**
(DOCX)

Table S9 **Logistic regression analysis of self-reported diabetes, *excluding* subjects with [BPA] > 80.1 ng/ml, per standard deviation increase of Bisphenol A exposure for NHANES 03-04 (N = 1,455), 05-06 (N = 1,498), 07-08 (N = 1,705), and a pooled sample (N = 4,658).**
(DOCX)

Table S10 **Logistic regression analysis of self-reported diabetes, *excluding* subjects [BPA]<LLOD and >99^th^ percentile, per standard deviation increase of Bisphenol A exposure for NHANES 03-04 (N = 1,455), 05-06 (N = 1,498), 07-08 (N = 1,705), and a pooled sample (N = 4,658).**
(DOCX)

Table S11 **Log-linear analysis of self-reported diabetes, *excluding* subjects with [BPA] > 80.1 ng/ml, per ten-fold increase in Bisphenol A exposure, or doubling of log(BPA), for NHANES 03-04 (N = 1,455), 05-06 (N = 1,498), 07-08 (N = 1,705), and a pooled sample (N = 4,658).**
(DOCX)

Table S12 **Log-linear analysis of self-reported diabetes, *excluding* subjects [BPA]<LLOD and >99^th^ percentile, per ten-fold increase in Bisphenol A exposure, or doubling of log(BPA), for NHANES 03-04 (N = 1,455), 05-06 (N = 1,498), 07-08 (N = 1,705), and a pooled sample (N = 4,658).**
(DOCX)

Table S13 **Akaike information criterion (AIC) and Bayesian information criterion (BIC) across various functional-forms and inclusion/exclusion criteria for CHD in the pooled data.**
(DOCX)

Table S14 **Akaike information criterion (AIC) and Bayesian information criterion (BIC) across various functional-forms and inclusion/exclusion criteria for diabetes in the pooled data.**
(DOCX)

Table S15 **Dose-response regression analysis of self-reported CHD for NHANES 03-04 (N = 1,455), 05-06 (N = 1,498), 07-08 (N = 1,705), and a pooled sample (N = 4,658).**
(DOCX)

Table S16 **Dose-response regression analysis of self-reported diabetes for NHANES 03-04 (N = 1,455), 05-06 (N = 1,498), 07-08 (N = 1,705), and a pooled sample (N = 4,658).**
(DOCX)

Table S17 **Marginal effects for logistic regression model in the analysis of self-reported CHD.**
(DOCX)

Table S18 **Marginal effects for logistic regression model in the analysis of self-reported diabetes.**
(DOCX)

Table S19 **Marginal effects for log-linear logistic regression model in the analysis of self-reported CHD.**
(DOCX)

Table S20 **Marginal effects for log-linear logistic regression model in the analysis of self-reported diabetes.**
(DOCX)

Table S21 **Marginal effects for dose-response regression model for the pooled data.**
(DOCX)

Table S22 **Main effect, with interacted coefficients by year, and marginal effects for CHD on Bisphenol A over various functional forms, in the pooled data using model 5 only (all covariates except for phthalates).** Marginal effects are reported at the means of all covariates.
(DOCX)

Table S23 **Main effect, with interacted coefficients by year, and marginal effects for Diabetes on Bisphenol A over various functional forms, in the pooled data using model 5 only (all covariates except for phthalates).** Marginal effects are reported at the means of all covariates.
(DOCX)
