# Supplementary material for: Disconcordance in Statistical Models of Bisphenol A and Chronic Disease Outcomes in NHANES 2003-08
Source: PLoS One. 2013 Nov 6;8(11):e79944. doi: 10.1371/journal.pone.0079944 (PMC3819299; doi:10.1371/journal.pone.0079944)
Supplement: Table S1 — Sociodemographic variables included in Model 3 and their unadjusted correlations with BPA. (DOCX) [file pone.0079944.s001.docx]

Table S1. Sociodemographic variables included in Model 3 and their unadjusted correlations with BPA.

|  |  | **03-04** | **05-06** | **07-08** | **pooled** |
| --- | --- | --- | --- | --- | --- |
|  |  | **coef. (p-value)** | **coef. (p-value)** | **coef. (p-value)** | **coef. (p-value)** |
| Veteran/Military Status | Yes | 0 (ref) | 0 (ref) | 0 (ref) | 0 (ref) |
| (dmqmilit)* | No | 0.009 (0.989) | 0.702 (0.2) | -0.502 (0.697) | 0.101 (0.839) |
|  |  |  |  |  |  |
| Citizenship Status | By birth or naturalization | 0 (ref) | 0 (ref) | 0 (ref) | 0 (ref) |
| (dmdcitzn)* | Not a citizen | -0.533 (0.259) | 0.475 (0.676) | -0.4 (0.562) | -0.145 (0.762) |
|  | Unknown | NA | -0.048 (0.888) | NA | -0.513 (0.013) |
|  |  |  |  |  |  |
| Marital Status | Married/Living w. Partner | 0 (ref) | 0 (ref) | 0 (ref) | 0 (ref) |
| (dmdmartl)* | Widowed | -2.001 (<0.001) | -0.001 (0.999) | -0.473 (0.655) | -0.739 (0.201) |
|  | Divorced/Separated | 0.679 (0.188) | -0.04 (0.939) | 0.299 (0.639) | 0.299 (0.348) |
|  | Never married/unknown | 1.658 (0.005) | 0.845 (0.263) | 0.711 (0.376) | 1.094 (0.01) |
|  |  |  |  |  |  |
| Household Size | 1 | 0 (ref) | 0 (ref) | 0 (ref) | 0 (ref) |
| (dmdhhsiz)* | 2 | -0.654 (0.359) | -0.608 (0.238) | -0.924 (0.199) | -0.668 (0.082) |
|  | 3 | -0.565 (0.487) | 0.145 (0.836) | -0.015 (0.989) | -0.129 (0.805) |
|  | 4 | -0.499 (0.514) | 1.9 (0.144) | -1.431 (0.139) | -0.027 (0.962) |
|  | 5+ | 0.124 (0.881) | 2.014 (0.047) | -0.396 (0.693) | 0.626 (0.252) |
|  |  |  |  |  |  |
| Pregnancy Status | Yes | 1.206 (0.291) | -1.324 (0.143) | -1.324 (0.143) | -0.081 (0.897) |
| (ridexprg)* | No | 1.717 (<0.001) | 0.153 (0.852) | 0.153 (0.852) | 0.817 (0.089) |
|  | Unknown | 0 (ref) | 0 (ref) | 0 (ref) | 0 (ref) |
|  |  |  |  |  |  |
| Language of subject Interview | English | 0 (ref) | 0 (ref) | 0 (ref) | 0 (ref) |
| (sialang)* | Spanish | -0.059 (0.939) | 2.116 (0.086) | -1.213 (0.022) | 0.188 (0.721) |
|  |  |  |  |  |  |
| Covered by Health Insurance | Yes | 0 (ref) | 0 (ref) | 0 (ref) | 0 (ref) |
| (hid010)* | No/Unknown | 1.173 (0.03) | 1.06 (0.058) | 1.047 (0.065) | 1.087 (0.001) |
|  |  |  |  |  |  |
| Did you work last week? | Yes | 0 (ref) | 0 (ref) | 0 (ref) | 0 (ref) |
| (ocd150)* | No | 0.33 (0.437) | 0.204 (0.748) | -0.274 (0.561) | 0.136 (0.651) |

NA - not applicable because no observations feel in this category for the specified survey cycle

*NHANES variable identifier
