# Supplementary material for: Disconcordance in Statistical Models of Bisphenol A and Chronic Disease Outcomes in NHANES 2003-08
Source: PLoS One. 2013 Nov 6;8(11):e79944. doi: 10.1371/journal.pone.0079944 (PMC3819299; doi:10.1371/journal.pone.0079944)
Supplement: Table S2 — Model 4 variables, sources of BPA exposure, and unadjusted correlations with BPA. (DOCX) [file pone.0079944.s002.docx]

Table S2. Model 4 variables, sources of BPA exposure, and unadjusted correlations with BPA.

|  |  | **03-04** | **05-06** | **07-08** | **Pooled** |
| --- | --- | --- | --- | --- | --- |
|  |  | **coef. (p-value)** | **coef. (p-value)** | **coef. (p-value)** | **coef. (p-value)** |
| Consumption of Bottled H2O (past 24hrs) | 1-50 percentile | 0 (ref) | 0 (ref) | 0 (ref) | 0 (ref) |
| (dr1bwatr)* | 51-75 percentile | 0.609 (0.514) | 0.243 (0.82) | 1.009 (0.027) | 0.618 (0.203) |
|  | 76-100 percentile | 0.243 (0.876) | -0.961 (0.378) | 0.608 (0.315) | 0.138 (0.848) |
|  | Unknown | 0.155 (0.898) | -0.041 (0.968) | 1.618 (0.043) | 0.558 (0.339) |
|  |  |  |  |  |  |
| Consumption of alcohol (past year) | 1-25 percentile | 0 (ref) | 0 (ref) | 0 (ref) | 0 (ref) |
| (alq120q, alq120u)* | 26-50 percentile | -1.972 (0.096) | -0.295 (0.801) | 0.715 (0.581) | -0.441 (0.56) |
|  | 51-75 percentile | -0.979 (0.375) | -0.745 (0.425) | 0.451 (0.534) | -0.372 (0.514) |
|  | 76-100 percentile | -1.499 (0.18) | -1.078 (0.247) | -0.672 (0.211) | -1.042 (0.062) |
|  | Unknown | -0.39 (0.681) | 0.471 (0.673) | -0.416 (0.41) | -0.068 (0.898) |
|  |  |  |  |  |  |
| Consumption of tunafish (past year) | None | 0 (ref) | 0 (ref) | 0 (ref) | 0 (ref) |
| (drd370bq)* | 1-50 percentile | 0.961 (0.251) | -0.907 (0.304) | 0.447 (0.483) | -0.225 (0.564) |
|  | 51-100 percentile | 0.195 (0.698) | -1.1 (0.246) | 0.483 (0.254) | -0.387 (0.349) |
|  | Unknown | -0.714 (0.512) | -1.907 (0.014) | 0.802 (0.51) | -0.83 (0.128) |

* - NHANES variable identifier
