# Supplementary material for: Disconcordance in Statistical Models of Bisphenol A and Chronic Disease Outcomes in NHANES 2003-08
Source: PLoS One. 2013 Nov 6;8(11):e79944. doi: 10.1371/journal.pone.0079944 (PMC3819299; doi:10.1371/journal.pone.0079944)
Supplement: Table S3 — Model 5 variables, healthy behaviours, and unadjusted correlations with BPA. (DOCX) [file pone.0079944.s003.docx]

Table S3. Model 5 variables, healthy behaviours, and unadjusted correlations with BPA.

|  |  | **03-04** | **05-06** | **07-08** | **Pooled** |
| --- | --- | --- | --- | --- | --- |
|  |  | **coef. (p-value)** | **coef. (p-value)** | **coef. (p-value)** | **coef. (p-value)** |
| Have emotional support? | Yes | 0 (ref) | 0 (ref) | 0 (ref) | 0 (ref) |
| (ssq011)* | No/Does not need | 0.341 (0.737) | -1.314 (0.024) | -0.478 (0.708) | -0.441 (0.485) |
|  | Not asked | 0.891 (0.081) | 0.595 (0.249) | 0.401 (0.509) | 0.633 (0.05) |
|  | Unknown | NA | -0.975 (0.045) | NA | -1.342 (<0.001) |
|  |  |  |  |  |  |
| Are you dieting? | Yes | 0 (ref) | 0 (ref) | 0 (ref) | 0 (ref) |
| (drqsdiet)* | No/Unknown | 0.198 (0.572) | -0.771 (0.53) | 1.35 (0.027) | 0.214 (0.673) |
|  |  |  |  |  |  |
| Use a water treatment device? | Yes | 0 (ref) | 0 (ref) | 0 (ref) | 0 (ref) |
| (hoq080)* | No/Unknown | 0.6 (0.257) | 1.648 (<0.001) | -0.212 (0.801) | 0.685 (0.065) |
|  |  |  |  |  |  |
| Routine source for healthcare? | Yes | 0 (ref) | 0 (ref) | 0 (ref) | 0 (ref) |
| (huq030)* | No/Unknown | 0.022 (0.957) | 0.422 (0.597) | 0.406 (0.464) | 0.291 (0.415) |
|  |  |  |  |  |  |
| Vaccinated for Hepatitis A or B? | Yes | 0 (ref) | 0 (ref) | 0 (ref) | 0 (ref) |
| (imd010, imd010)* | Some doses, but not all | 0.006 (0.995) | -0.192 (0.795) | -0.192 (0.795) | -0.206 (0.641) |
|  | No | 0.07 (0.838) | 0.151 (0.84) | 0.151 (0.84) | 0.29 (0.396) |
|  | Unknown | 0.598 (0.438) | 1.621 (0.146) | 1.621 (0.146) | 0.959 (0.097) |
|  |  |  |  |  |  |
| Taking any dietary supplements? | Yes | 0 (ref) | 0 (ref) | 0 (ref) | 0 (ref) |
| (dsd010)* | No/Unknown | 1.14 (0.003) | 0.185 (0.705) | 0.446 (0.625) | 0.567 (0.127) |
|  |  |  |  |  |  |
| Can’t afford balanced meals? | Often/Sometimes/Unknown | 0 (ref) | 0 (ref) | 0 (ref) | 0 (ref) |
| (fsd032c)* | Never true/Screened out | -0.616 (0.232) | -0.743 (0.246) | -0.546 (0.517) | -0.687 (0.073) |

NA - not applicable because no observations feel in this category for the specified survey cycle

* - NHANES variable name
