# Supplementary material for: Disconcordance in Statistical Models of Bisphenol A and Chronic Disease Outcomes in NHANES 2003-08
Source: PLoS One. 2013 Nov 6;8(11):e79944. doi: 10.1371/journal.pone.0079944 (PMC3819299; doi:10.1371/journal.pone.0079944)
Supplement: Table S4 — Biomarker, creatinine and phthalate, and unadjusted correlations with BPA. (DOCX) [file pone.0079944.s004.docx]

Table S4. Biomarker, creatinine and phthalate, and unadjusted correlations with BPA.

|  | **03-04** | **05-06** | **07-08** | **Pooled** |
| --- | --- | --- | --- | --- |
|  | **coef. (p-value)** | **coef. (p-value)** | **coef. (p-value)** | **coef. (p-value)** |
| Urinary Creatinine (mg/dl) | 0.029 (<0.001) | 0.018 (<0.001) | 0.023 (<0.001) | 0.024 (<0.001) |
| Mono(2-ethylhexyl) phthalate (ng/ml) † | NA | 0.003 (0.158) | 0.013 (0.117) | 0.009 (0.079) |
| Mono-isobutyl phthalate (ng/ml) † | NA | 0.028 (0.047) | 0.045 (<0.001) | 0.036 (0.002) |
| Mono-n-butyl phthalate (ng/ml) † | NA | 0.008 (0.147) | 0.003 (0.127) | 0.004 (0.091) |

†Phthalate data was only pooled in the two cycles with available exposure data.
