# Supplementary material for: Disconcordance in Statistical Models of Bisphenol A and Chronic Disease Outcomes in NHANES 2003-08
Source: PLoS One. 2013 Nov 6;8(11):e79944. doi: 10.1371/journal.pone.0079944 (PMC3819299; doi:10.1371/journal.pone.0079944)
Supplement: Table S5 — Logistic regression analysis of self-reported CHD, excluding subjects with [BPA] > 80.1 ng/ml, per standard deviation increase of Bisphenol A exposure for NHANES 03-04 (N = 1,455), 05-06 (N = 1,498), 07-08 (N = 1,705), and a pooled sample (N = 4,658). (DOCX) [file pone.0079944.s005.docx]

Table S5. Logistic regression analysis of self-reported CHD, *excluding* subjects with [BPA] > 80.1 ng/ml, per standard deviation increase of Bisphenol A exposure for NHANES 03-04 (N = 1,455), 05-06 (N = 1,498), 07-08 (N = 1,705), and a pooled sample (N = 4,658).

|  | NHANES 03-04 | | NHANES 05-06 | | NHANES 07-08 | | Pooled |  |
| --- | --- | --- | --- | --- | --- | --- | --- | --- |
|  | OR (95% CI) | | OR (95% CI) | | OR (95% CI) | | OR (95% CI) | |
| Model 1 | 1.408* | (1.093 - 1.815) | 1.575 | (0.864 - 2.870) | 1.209 | (0.957 - 1.527) | 1.447** | (1.159 - 1.805) |
| Model 2 | 1.596* | (1.126 - 2.262) | 1.848 | (1.074 - 3.182) | 1.222 | (0.935 - 1.599) | 1.471** | (1.180 - 1.835) |
| Model 3 | 1.713** | (1.180 - 2.486) | 2.206** | (1.246 - 3.906) | 1.274 | (0.961 - 1.690) | 1.470** | (1.183 - 1.828) |
| Model 4 | 1.855** | (1.248 - 2.758) | 2.245** | (1.258 - 4.007) | 1.361 | (0.995 - 1.864) | 1.490** | (1.214 - 1.830) |
| Model 5 | 1.824** | (1.288 - 2.583) | 1.923** | (1.200 - 3.082) | 1.474* | (1.101 - 1.972) | 1.498** | (1.223 - 1.834) |
| Model 6 | -- | -- | 2.213** | (1.281 - 3.822) | 1.560** | (1.176 - 2.070) | -- | -- |

* - p < 0.025 ; ** - p < 0.01

Model 1: adjusted for age, sex, and urinary creatinine concentration

Model 2: further adjusted for race/ethnicity, income, smoking, body mass index, and waist circumference

Model 3: veteran/military status, citizenship status, marital status, household size, pregnancy status, language at subject interview, health insurance coverage, and employment status in the prior week

Model 4: consumption of bottled water in the past 24 hrs, consumption of alcohol, and annual consumption of tuna fish

Model 5: presence of emotional support in one’s life, being on a diet, using a water treatment device, access to a routine source of health care, vaccinated for Hepatitis A or B, consumption of dietary supplements (vitamins or minerals), and inability to purchase balanced meals on a consistent basis

Model 6: concentration of (2-ethylhexyl) phthalate (MEHP), mono-isobutyl phthalate (MiBP), and mono-n-butyl phthalate (MeBP)
