# Supplementary material for: Disconcordance in Statistical Models of Bisphenol A and Chronic Disease Outcomes in NHANES 2003-08
Source: PLoS One. 2013 Nov 6;8(11):e79944. doi: 10.1371/journal.pone.0079944 (PMC3819299; doi:10.1371/journal.pone.0079944)
Supplement: Table S6 — Logistic regression analysis of self-reported CHD, excluding subjects [BPA]99th percentile, per standard deviation increase of Bisphenol A exposure for NHANES 03-04 (N = 1,455), 05-06 (N = 1,498), 07-08 (N = 1,705), and a pooled sample (N = 4,658). (DOCX) [file pone.0079944.s006.docx]

Table S6. Logistic regression analysis of self-reported CHD, *excluding* subjects [BPA]<LLOD and >99^th^ percentile, per standard deviation increase of Bisphenol A exposure for NHANES 03-04 (N = 1,455), 05-06 (N = 1,498), 07-08 (N = 1,705), and a pooled sample (N = 4,658).

|  | NHANES 03-04 | | NHANES 05-06 | | NHANES 07-08 | | Pooled |  |
| --- | --- | --- | --- | --- | --- | --- | --- | --- |
|  | OR (95% CI) | | OR (95% CI) | | OR (95% CI) | | OR (95% CI) | |
| Model 1 | 1.520 | (0.637 - 3.628) | 1.754 | (0.586 - 5.256) | 1.569** | (1.146 - 2.149) | 1.735* | (1.125 - 2.675) |
| Model 2 | 2.003 | (0.908 - 4.419) | 2.177 | (0.862 - 5.495) | 1.596* | (1.078 - 2.364) | 1.830** | (1.184 - 2.829) |
| Model 3 | 2.095* | (1.148 - 3.824) | 2.515* | (1.043 - 6.065) | 1.560* | (1.043 - 2.333) | 1.805** | (1.166 - 2.795) |
| Model 4 | 1.892 | (0.961 - 3.725) | 3.284 | (0.830 - 13.00) | 1.577* | (1.060 - 2.346) | 1.860** | (1.201 - 2.882) |
| Model 5 | 2.030 | (0.885 - 4.657) | 3.136 | (0.602 - 16.34) | 1.831** | (1.280 - 2.620) | 1.940** | (1.224 - 3.075) |
| Model 6 | -- | -- | 3.782 | (0.787 - 18.17) | 1.829** | (1.285 - 2.603) | -- | -- |

* - p < 0.025 ; ** - p < 0.01

Model 1: adjusted for age, sex, and urinary creatinine concentration

Model 2: further adjusted for race/ethnicity, income, smoking, body mass index, and waist circumference

Model 3: veteran/military status, citizenship status, marital status, household size, pregnancy status, language at subject interview, health insurance coverage, and employment status in the prior week

Model 4: consumption of bottled water in the past 24 hrs, consumption of alcohol, and annual consumption of tuna fish

Model 5: presence of emotional support in one’s life, being on a diet, using a water treatment device, access to a routine source of health care, vaccinated for Hepatitis A or B, consumption of dietary supplements (vitamins or minerals), and inability to purchase balanced meals on a consistent basis

Model 6: concentration of (2-ethylhexyl) phthalate (MEHP), mono-isobutyl phthalate (MiBP), and mono-n-butyl phthalate (MeBP)
