# Supplementary material for: Disconcordance in Statistical Models of Bisphenol A and Chronic Disease Outcomes in NHANES 2003-08
Source: PLoS One. 2013 Nov 6;8(11):e79944. doi: 10.1371/journal.pone.0079944 (PMC3819299; doi:10.1371/journal.pone.0079944)
Supplement: Table S7 — Log-linear analysis of self-reported CHD, excluding subjects with [BPA] > 80.1 ng/ml, per ten-fold increase in Bisphenol A exposure for NHANES 03-04 (N = 1,455), 05-06 (N = 1,498), 07-08 (N = 1,705), and a pooled sample (N = 4,658). (DOCX) [file pone.0079944.s007.docx]

Table S7. Log-linear analysis of self-reported CHD, *excluding* subjects with [BPA] > 80.1 ng/ml, per tenfold increase of Bisphenol A exposure for NHANES 03-04 (N = 1,455), 05-06 (N = 1,498), 07-08 (N = 1,705), and a pooled sample (N = 4,658).

|  | NHANES 03-04 | | NHANES 05-06 | | NHANES 07-08 | | Pooled |  |
| --- | --- | --- | --- | --- | --- | --- | --- | --- |
|  | OR (95% CI) | | OR (95% CI) | | OR (95% CI) | | OR (95% CI) | |
| Model 1 | 1.527 | (0.918 - 2.542) | 1.139 | (0.693 - 1.874) | 1.480 | (0.968 - 2.261) | 1.346* | (1.042 - 1.739) |
| Model 2 | 1.860 | (1.023 - 3.383) | 1.129 | (0.738 - 1.727) | 1.523 | (1.011 - 2.294) | 1.345* | (1.048 - 1.726) |
| Model 3 | 1.815* | (1.155 - 2.852) | 1.172 | (0.789 - 1.743) | 1.628* | (1.080 - 2.453) | 1.346* | (1.056 - 1.716) |
| Model 4 | 1.661** | (1.184 - 2.331) | 1.237 | (0.773 - 1.980) | 1.679* | (1.097 - 2.570) | 1.330 | (1.028 - 1.721) |
| Model 5 | 1.584 | (1.066 - 2.354) | 1.182 | (0.762 - 1.833) | 1.731* | (1.105 - 2.711) | 1.311 | (1.015 - 1.694) |
| Model 6 | -- | -- | 1.469 | (0.888 - 2.430) | 1.757 | (1.071 - 2.881) | -- | -- |

* - p < 0.025 ; ** - p < 0.01

Model 1: adjusted for age, sex, and urinary creatinine concentration

Model 2: further adjusted for race/ethnicity, income, smoking, body mass index, and waist circumference

Model 3: veteran/military status, citizenship status, marital status, household size, pregnancy status, language at subject interview, health insurance coverage, and employment status in the prior week

Model 4: consumption of bottled water in the past 24 hrs, consumption of alcohol, and annual consumption of tuna fish

Model 5: presence of emotional support in one’s life, being on a diet, using a water treatment device, access to a routine source of health care, vaccinated for Hepatitis A or B, consumption of dietary supplements (vitamins or minerals), and inability to purchase balanced meals on a consistent basis

Model 6: concentration of (2-ethylhexyl) phthalate (MEHP), mono-isobutyl phthalate (MiBP), and mono-n-butyl phthalate (MeBP)
