# Supplementary material for: Disconcordance in Statistical Models of Bisphenol A and Chronic Disease Outcomes in NHANES 2003-08
Source: PLoS One. 2013 Nov 6;8(11):e79944. doi: 10.1371/journal.pone.0079944 (PMC3819299; doi:10.1371/journal.pone.0079944)
Supplement: Table S8 — Log-linear analysis of self-reported CHD, excluding subjects [BPA]99th percentile, per ten-fold increase in Bisphenol A exposure for NHANES 03-04 (N = 1,455), 05-06 (N = 1,498), 07-08 (N = 1,705), and a pooled sample (N = 4,658). (DOCX) [file pone.0079944.s008.docx]

Table S8. Log-linear analysis of self-reported CHD, *excluding* subjects [BPA]<LLOD and >99^th^ percentile, per tenfold increase of Bisphenol A exposure for NHANES 03-04 (N = 1,455), 05-06 (N = 1,498), 07-08 (N = 1,705), and a pooled sample (N = 4,658).

|  | NHANES 03-04 | | NHANES 05-06 | | NHANES 07-08 | | Pooled |  |
| --- | --- | --- | --- | --- | --- | --- | --- | --- |
|  | OR (95% CI) | | OR (95% CI) | | OR (95% CI) | | OR (95% CI) | |
| Model 1 | 1.290 | (0.621 - 2.682) | 1.253 | (0.639 - 2.457) | 1.597* | (1.068 - 2.389) | 1.385 | (1.009 - 1.901) |
| Model 2 | 1.551 | (0.756 - 3.181) | 1.322 | (0.694 - 2.518) | 1.732** | (1.241 - 2.419) | 1.403 | (1.027 - 1.915) |
| Model 3 | 1.535 | (0.931 - 2.530) | 1.356 | (0.696 - 2.645) | 1.777** | (1.217 - 2.594) | 1.393 | (1.025 - 1.892) |
| Model 4 | 1.488 | (0.879 - 2.522) | 1.469 | (0.655 - 3.293) | 1.853** | (1.334 - 2.573) | 1.402 | (1.021 - 1.926) |
| Model 5 | 1.429 | (0.772 - 2.645) | 1.446 | (0.548 - 3.816) | 2.060** | (1.459 - 2.906) | 1.376 | (0.985 - 1.921) |
| Model 6 | -- | -- | 1.899 | (0.722 - 4.993) | 2.122** | (1.479 - 3.045) | -- | -- |

* - p < 0.025 ; ** - p < 0.01

Model 1: adjusted for age, sex, and urinary creatinine concentration

Model 2: further adjusted for race/ethnicity, income, smoking, body mass index, and waist circumference

Model 3: veteran/military status, citizenship status, marital status, household size, pregnancy status, language at subject interview, health insurance coverage, and employment status in the prior week

Model 4: consumption of bottled water in the past 24 hrs, consumption of alcohol, and annual consumption of tuna fish

Model 5: presence of emotional support in one’s life, being on a diet, using a water treatment device, access to a routine source of health care, vaccinated for Hepatitis A or B, consumption of dietary supplements (vitamins or minerals), and inability to purchase balanced meals on a consistent basis

Model 6: concentration of (2-ethylhexyl) phthalate (MEHP), mono-isobutyl phthalate (MiBP), and mono-n-butyl phthalate (MeBP)
