# Supplementary material for: Disconcordance in Statistical Models of Bisphenol A and Chronic Disease Outcomes in NHANES 2003-08
Source: PLoS One. 2013 Nov 6;8(11):e79944. doi: 10.1371/journal.pone.0079944 (PMC3819299; doi:10.1371/journal.pone.0079944)
Supplement: Table S9 — Logistic regression analysis of self-reported diabetes, excluding subjects with [BPA] > 80.1 ng/ml, per standard deviation increase of Bisphenol A exposure for NHANES 03-04 (N = 1,455), 05-06 (N = 1,498), 07-08 (N = 1,705), and a pooled sample (N = 4,658). (DOCX) [file pone.0079944.s009.docx]

Table S9. Logistic regression analysis of self-reported diabetes, *excluding* subjects with [BPA] > 80.1 ng/ml, per standard deviation increase of Bisphenol A exposure for NHANES 03-04 (N = 1,455), 05-06 (N = 1,498), 07-08 (N = 1,705), and a pooled sample (N = 4,658).

|  | NHANES 03-04 | | NHANES 05-06 | | NHANES 07-08 | | Pooled |  |  |
| --- | --- | --- | --- | --- | --- | --- | --- | --- | --- |
|  | OR (95% CI) | | OR (95% CI) | | OR (95% CI) | | OR (95% CI) | |  |
| Model 1 | 1.404** | (1.210 - 1.628) | 1.164 | (0.719 - 1.884) | 0.724 | (0.522 - 1.003) | 1.202 | (1.012 - 1.426) | |
| Model 2 | 1.400** | (1.245 - 1.574) | 1.062 | (0.553 - 2.039) | 0.711 | (0.504 - 1.002) | 1.207* | (1.027 - 1.417) | |
| Model 3 | 1.358** | (1.237 - 1.491) | 1.249 | (0.616 - 2.531) | 0.711 | (0.493 - 1.025) | 1.214* | (1.037 - 1.421) | |
| Model 4 | 1.359** | (1.187 - 1.556) | 1.264 | (0.633 - 2.526) | 0.712 | (0.484 - 1.049) | 1.211* | (1.035 - 1.417) | |
| Model 5 | 1.398** | (1.183 - 1.653) | 1.307 | (0.674 - 2.538) | 0.717 | (0.496 - 1.038) | 1.235** | (1.057 - 1.443) | |
| Model 6 | -- | -- | 1.304 | (0.670 - 2.537) | 0.700 | (0.460 - 1.065) | -- | -- |  |

* - p < 0.025 ; ** - p < 0.01

Model 1: adjusted for age, sex, and urinary creatinine concentration

Model 2: further adjusted for race/ethnicity, income, smoking, body mass index, and waist circumference

Model 3: veteran/military status, citizenship status, marital status, household size, pregnancy status, language at subject interview, health insurance coverage, and employment status in the prior week

Model 4: consumption of bottled water in the past 24 hrs, consumption of alcohol, and annual consumption of tuna fish

Model 5: presence of emotional support in one’s life, being on a diet, using a water treatment device, access to a routine source of health care, vaccinated for Hepatitis A or B, consumption of dietary supplements (vitamins or minerals), and inability to purchase balanced meals on a consistent basis

Model 6: concentration of (2-ethylhexyl) phthalate (MEHP), mono-isobutyl phthalate (MiBP), and mono-n-butyl phthalate (MeBP)
