# Supplementary material for: Disconcordance in Statistical Models of Bisphenol A and Chronic Disease Outcomes in NHANES 2003-08
Source: PLoS One. 2013 Nov 6;8(11):e79944. doi: 10.1371/journal.pone.0079944 (PMC3819299; doi:10.1371/journal.pone.0079944)
Supplement: Table S10 — Logistic regression analysis of self-reported diabetes, excluding subjects [BPA]99th percentile, per standard deviation increase of Bisphenol A exposure for NHANES 03-04 (N = 1,455), 05-06 (N = 1,498), 07-08 (N = 1,705), and a pooled sample (N = 4,658). (DOCX) [file pone.0079944.s010.docx]

Table S10. Logistic regression analysis of self-reported diabetes, *excluding* subjects [BPA]<LLOD and >99^th^ percentile, per standard deviation increase of Bisphenol A exposure for NHANES 03-04 (N = 1,455), 05-06 (N = 1,498), 07-08 (N = 1,705), and a pooled sample (N = 4,658).

|  | NHANES 03-04 | | NHANES 05-06 | | NHANES 07-08 | | Pooled |  |
| --- | --- | --- | --- | --- | --- | --- | --- | --- |
|  | OR (95% CI) | | OR (95% CI) | | OR (95% CI) | | OR (95% CI) | |
| Model 1 | 1.475** | (1.142 - 1.903) | 1.806 | (0.677 - 4.822) | 0.748 | (0.491 - 1.139) | 1.288 | (0.958 - 1.732) |
| Model 2 | 1.445** | (1.127 - 1.851) | 1.580 | (0.465 - 5.376) | 0.723 | (0.465 - 1.124) | 1.255 | (0.908 - 1.735) |
| Model 3 | 1.415* | (1.099 - 1.823) | 1.681 | (0.469 - 6.024) | 0.727 | (0.451 - 1.172) | 1.217 | (0.859 - 1.725) |
| Model 4 | 1.350 | (1.032 - 1.767) | 2.074 | (0.572 - 7.518) | 0.731 | (0.449 - 1.190) | 1.247 | (0.883 - 1.762) |
| Model 5 | 1.414 | (1.035 - 1.933) | 2.212 | (0.615 - 7.954) | 0.761 | (0.476 - 1.216) | 1.301 | (0.913 - 1.853) |
| Model 6 | -- | -- | 2.203 | (0.600 - 8.084) | 0.735 | (0.466 - 1.159) | -- | -- |

* - p < 0.025 ; ** - p < 0.01

Model 1: adjusted for age, sex, and urinary creatinine concentration

Model 2: further adjusted for race/ethnicity, income, smoking, body mass index, and waist circumference

Model 3: veteran/military status, citizenship status, marital status, household size, pregnancy status, language at subject interview, health insurance coverage, and employment status in the prior week

Model 4: consumption of bottled water in the past 24 hrs, consumption of alcohol, and annual consumption of tuna fish

Model 5: presence of emotional support in one’s life, being on a diet, using a water treatment device, access to a routine source of health care, vaccinated for Hepatitis A or B, consumption of dietary supplements (vitamins or minerals), and inability to purchase balanced meals on a consistent basis

Model 6: concentration of (2-ethylhexyl) phthalate (MEHP), mono-isobutyl phthalate (MiBP), and mono-n-butyl phthalate (MeBP)
