# Supplementary material for: Disconcordance in Statistical Models of Bisphenol A and Chronic Disease Outcomes in NHANES 2003-08
Source: PLoS One. 2013 Nov 6;8(11):e79944. doi: 10.1371/journal.pone.0079944 (PMC3819299; doi:10.1371/journal.pone.0079944)
Supplement: Table S11 — Log-linear analysis of self-reported diabetes, excluding subjects with [BPA] > 80.1 ng/ml, per ten-fold increase in Bisphenol A exposure, or doubling of log(BPA), for NHANES 03-04 (N = 1,455), 05-06 (N = 1,498), 07-08 (N = 1,705), and a pooled sample (N = 4,658). (DOCX) [file pone.0079944.s011.docx]

Table S11. Log-linear analysis of self-reported diabetes, *excluding* subjects with [BPA] > 80.1 ng/ml, per ten-fold increase in Bisphenol A exposure, or doubling of log(BPA), for NHANES 03-04 (N = 1,455), 05-06 (N = 1,498), 07-08 (N = 1,705), and a pooled sample (N = 4,658).

|  | NHANES 03-04 | | NHANES 05-06 | | NHANES 07-08 | | Pooled |  |
| --- | --- | --- | --- | --- | --- | --- | --- | --- |
|  | OR (95% CI) | | OR (95% CI) | | OR (95% CI) | | OR (95% CI) | |
| Model 1 | 1.562** | (1.323 - 1.844) | 1.244 | (0.943 - 1.641) | 0.979 | (0.804 - 1.193) | 1.237** | (1.089 - 1.405) |
| Model 2 | 1.548** | (1.315 - 1.823) | 1.191 | (0.823 - 1.723) | 0.979 | (0.821 - 1.167) | 1.214* | (1.048 - 1.406) |
| Model 3 | 1.524** | (1.295 - 1.794) | 1.246 | (0.868 - 1.789) | 0.970 | (0.797 - 1.182) | 1.221** | (1.055 - 1.412) |
| Model 4 | 1.476** | (1.286 - 1.694) | 1.274 | (0.862 - 1.881) | 0.944 | (0.758 - 1.174) | 1.214* | (1.048 - 1.407) |
| Model 5 | 1.492** | (1.267 - 1.757) | 1.272 | (0.873 - 1.854) | 0.944 | (0.762 - 1.171) | 1.224** | (1.059 - 1.414) |
| Model 6 | -- | -- | 1.261 | (0.858 - 1.854) | 0.880 | (0.682 - 1.136) | -- | -- |

* - p < 0.025 ; ** - p < 0.01

Model 1: adjusted for age, sex, and urinary creatinine concentration

Model 2: further adjusted for race/ethnicity, income, smoking, body mass index, and waist circumference

Model 3: veteran/military status, citizenship status, marital status, household size, pregnancy status, language at subject interview, health insurance coverage, and employment status in the prior week

Model 4: consumption of bottled water in the past 24 hrs, consumption of alcohol, and annual consumption of tuna fish

Model 5: presence of emotional support in one’s life, being on a diet, using a water treatment device, access to a routine source of health care, vaccinated for Hepatitis A or B, consumption of dietary supplements (vitamins or minerals), and inability to purchase balanced meals on a consistent basis

Model 6: concentration of (2-ethylhexyl) phthalate (MEHP), mono-isobutyl phthalate (MiBP), and mono-n-butyl phthalate (MeBP)
