# Supplementary material for: Disconcordance in Statistical Models of Bisphenol A and Chronic Disease Outcomes in NHANES 2003-08
Source: PLoS One. 2013 Nov 6;8(11):e79944. doi: 10.1371/journal.pone.0079944 (PMC3819299; doi:10.1371/journal.pone.0079944)
Supplement: Table S12 — Log-linear analysis of self-reported diabetes, excluding subjects [BPA]99th percentile, per ten-fold increase in Bisphenol A exposure, or doubling of log(BPA), for NHANES 03-04 (N = 1,455), 05-06 (N = 1,498), 07-08 (N = 1,705), and a pooled sample (N = 4,658). (DOCX) [file pone.0079944.s012.docx]

Table S12. Log-linear analysis of self-reported diabetes, *excluding* subjects [BPA]<LLOD and >99^th^ percentile, per ten-fold increase in Bisphenol A exposure, or doubling of log(BPA), for NHANES 03-04 (N = 1,455), 05-06 (N = 1,498), 07-08 (N = 1,705), and a pooled sample (N = 4,658).

|  | NHANES 03-04 | | NHANES 05-06 | | NHANES 07-08 | | Pooled |  |
| --- | --- | --- | --- | --- | --- | --- | --- | --- |
|  | OR (95% CI) | | OR (95% CI) | | OR (95% CI) | | OR (95% CI) | |
| Model 1 | 1.416** | (1.166 - 1.719) | 1.393 | (0.959 - 2.025) | 0.980 | (0.718 - 1.337) | 1.245* | (1.045 - 1.483) |
| Model 2 | 1.366** | (1.123 - 1.661) | 1.353 | (0.849 - 2.154) | 0.973 | (0.704 - 1.345) | 1.220 | (1.013 - 1.470) |
| Model 3 | 1.332** | (1.096 - 1.618) | 1.383 | (0.900 - 2.126) | 0.972 | (0.685 - 1.380) | 1.214 | (1.005 - 1.466) |
| Model 4 | 1.290* | (1.038 - 1.603) | 1.499 | (0.940 - 2.391) | 0.942 | (0.663 - 1.338) | 1.225 | (1.008 - 1.489) |
| Model 5 | 1.319 | (1.022 - 1.702) | 1.508 | (0.950 - 2.394) | 0.957 | (0.676 - 1.355) | 1.232 | (1.009 - 1.503) |
| Model 6 | -- | -- | 1.451 | (0.922 - 2.285) | 0.903 | (0.643 - 1.269) | -- | -- |

* - p < 0.025 ; ** - p < 0.01

Model 1: adjusted for age, sex, and urinary creatinine concentration

Model 2: further adjusted for race/ethnicity, income, smoking, body mass index, and waist circumference

Model 3: veteran/military status, citizenship status, marital status, household size, pregnancy status, language at subject interview, health insurance coverage, and employment status in the prior week

Model 4: consumption of bottled water in the past 24 hrs, consumption of alcohol, and annual consumption of tuna fish

Model 5: presence of emotional support in one’s life, being on a diet, using a water treatment device, access to a routine source of health care, vaccinated for Hepatitis A or B, consumption of dietary supplements (vitamins or minerals), and inability to purchase balanced meals on a consistent basis

Model 6: concentration of (2-ethylhexyl) phthalate (MEHP), mono-isobutyl phthalate (MiBP), and mono-n-butyl phthalate (MeBP)
