# Supplementary material for: Disconcordance in Statistical Models of Bisphenol A and Chronic Disease Outcomes in NHANES 2003-08
Source: PLoS One. 2013 Nov 6;8(11):e79944. doi: 10.1371/journal.pone.0079944 (PMC3819299; doi:10.1371/journal.pone.0079944)
Supplement: Table S13 — Akaike information criterion (AIC) and Bayesian information criterion (BIC) across various functional-forms and inclusion/exclusion criteria for CHD in the pooled data. (DOCX) [file pone.0079944.s013.docx]

Table S13. Akaike information criterion (AIC) and Bayesian information criterion (BIC) across various functional-forms and inclusion/exclusion criteria for CHD in the pooled data.

|  |  | **Linear** | | **Log-Linear** | | **Dose-response** | |
| --- | --- | --- | --- | --- | --- | --- | --- |
|  |  | **AIC**† | **BIC**† | **AIC**† | **BIC**† | **AIC**† | **BIC**† |
| Full data set | Model 1 | 4.11 | 4.11 | 4.09 | 4.09 | 4.08 | 4.08 |
|  | Model 2 | 3.93 | 3.93 | 3.92 | 3.92 | 3.90 | 3.90 |
|  | Model 3 | 3.88 | 3.88 | 3.86 | 3.86 | 3.85 | 3.85 |
|  | Model 4 | 3.75 | 3.75 | 3.74 | 3.74 | 3.71 | 3.71 |
|  | Model 5 | 3.62 | 3.62 | 3.61 | 3.61 | 3.58 | 3.58 |
|  |  |  |  |  |  |  |  |
| Omit BPA>80.1 | Model 1 | 4.08 | 4.08 | 4.08 | 4.08 | 4.07 | 4.07 |
|  | Model 2 | 3.90 | 3.90 | 3.91 | 3.91 | 3.90 | 3.90 |
|  | Model 3 | 3.85 | 3.85 | 3.86 | 3.86 | 3.84 | 3.84 |
|  | Model 4 | 3.72 | 3.72 | 3.73 | 3.73 | 3.71 | 3.71 |
|  | Model 5 | 3.59 | 3.59 | 3.61 | 3.61 | 3.58 | 3.58 |
|  |  |  |  |  |  |  |  |
| Omit <LLOD and | Model 1 | 3.57 | 3.57 | 3.57 | 3.57 | 3.57 | 3.57 |
| >95th percentile | Model 2 | 3.42 | 3.42 | 3.42 | 3.42 | 3.42 | 3.42 |
|  | Model 3 | 3.36 | 3.36 | 3.36 | 3.36 | 3.36 | 3.36 |
|  | Model 4 | 3.23 | 3.23 | 3.24 | 3.24 | 3.22 | 3.22 |
|  | Model 5 | 3.09 | 3.09 | 3.10 | 3.10 | 3.09 | 3.09 |

†Observed order of magnitude, for all reported AIC and BIC, was 10^7^
