# Supplementary material for: Disconcordance in Statistical Models of Bisphenol A and Chronic Disease Outcomes in NHANES 2003-08
Source: PLoS One. 2013 Nov 6;8(11):e79944. doi: 10.1371/journal.pone.0079944 (PMC3819299; doi:10.1371/journal.pone.0079944)
Supplement: Table S14 — Akaike information criterion (AIC) and Bayesian information criterion (BIC) across various functional-forms and inclusion/exclusion criteria for diabetes in the pooled data. (DOCX) [file pone.0079944.s014.docx]

**Table S14.** Akaike information criterion (AIC) and Bayesian information criterion (BIC) across various functional-forms and inclusion/exclusion criteria for diabetes in the pooled data.

|  |  | **Linear** | | **Log-Linear** | | **Dose-response** | |
| --- | --- | --- | --- | --- | --- | --- | --- |
|  |  | **AIC** | **BIC** | **AIC** | **BIC** | **AIC** | **BIC** |
| Full data set | Model 1 | 10.5 | 10.5 | 10.5 | 10.5 | 10.4 | 10.4 |
|  | Model 2 | 9.63 | 9.63 | 9.60 | 9.60 | 9.58 | 9.58 |
|  | Model 3 | 9.50 | 9.50 | 9.47 | 9.47 | 9.45 | 9.45 |
|  | Model 4 | 9.28 | 9.28 | 9.25 | 9.25 | 9.24 | 9.24 |
|  | Model 5 | 8.86 | 8.8 | 8.83 | 8.83 | 8.83 | 8.83 |
|  |  |  |  |  |  |  |  |
| Omit BPA>80.1 | Model 1 | 10.5 | 10.5 | 10.4 | 10.4 | 10.4 | 10.4 |
|  | Model 2 | 9.61 | 9.61 | 9.59 | 9.59 | 9.58 | 9.58 |
|  | Model 3 | 9.48 | 9.48 | 9.46 | 9.46 | 9.45 | 9.45 |
|  | Model 4 | 9.26 | 9.26 | 9.24 | 9.24 | 9.23 | 9.23 |
|  | Model 5 | 8.83 | 8.83 | 8.82 | 8.82 | 8.82 | 8.82 |
|  |  |  |  |  |  |  |  |
| Omit <LLOD and | Model 1 | 9.41 | 9.41 | 9.39 | 9.39 | 9.37 | 9.37 |
| >95th percentile | Model 2 | 8.64 | 8.64 | 8.62 | 8.62 | 8.61 | 8.61 |
|  | Model 3 | 8.54 | 8.54 | 8.52 | 8.52 | 8.50 | 8.50 |
|  | Model 4 | 8.31 | 8.31 | 8.29 | 8.29 | 8.28 | 8.28 |
|  | Model 5 | 7.91 | 7.91 | 7.90 | 7.90 | 7.89 | 7.89 |

†Observed order of magnitude, for all reported AIC and BIC, was 10^7^
