# Supplementary material for: Disconcordance in Statistical Models of Bisphenol A and Chronic Disease Outcomes in NHANES 2003-08
Source: PLoS One. 2013 Nov 6;8(11):e79944. doi: 10.1371/journal.pone.0079944 (PMC3819299; doi:10.1371/journal.pone.0079944)
Supplement: Table S15 — Dose-response regression analysis of self-reported CHD for NHANES 03-04 (N = 1,455), 05-06 (N = 1,498), 07-08 (N = 1,705), and a pooled sample (N = 4,658). (DOCX) [file pone.0079944.s015.docx]

Table S15. Dose-response regression analysis of self-reported CHD for NHANES 03-04 (N = 1,455), 05-06 (N = 1,498), 07-08 (N = 1,705), and a pooled sample (N = 4,658).

|  |  | **NHANES 03-04** | | **NHANES 05-06** | | **NHANES 07-08** | | **Pooled** |  |
| --- | --- | --- | --- | --- | --- | --- | --- | --- | --- |
|  | **[BPA] (ng/ml)** | **OR (95% CI)** | | **OR (95% CI)** | | **OR (95% CI)** | | **OR (95% CI)** | |
| Model 1 | <1.1 | Ref | -- | Ref | -- | Ref | -- | Ref | -- |
|  | 1.2-2.2 | 0.931 | (0.242 - 3.585) | 0.549 | (0.154 - 1.949) | 0.730 | (0.239 - 2.229) | 0.663 | (0.346 - 1.272) |
|  | 2.3-4.2 | 1.310 | (0.593 - 2.894) | 0.821 | (0.284 - 2.377) | 1.697 | (0.433 - 6.655) | 1.118 | (0.606 - 2.066) |
|  | >4.2 | 1.539 | (0.576 - 4.113) | 1.507 | (0.380 - 5.967) | 2.832 | (0.698 - 11.50) | 1.771 | (0.875 - 3.583) |
|  |  |  |  |  |  |  |  |  |  |
| Model 2 | <1.1 | Ref | -- | Ref | -- | Ref | -- | Ref | -- |
|  | 1.2-2.2 | 0.763 | (0.169 - 3.438) | 0.444 | (0.145 - 1.356) | 0.620 | (0.180 - 2.143) | 0.617 | (0.331 - 1.149) |
|  | 2.3-4.2 | 1.511 | (0.481 - 4.748) | 0.758 | (0.297 - 1.933) | 1.788 | (0.527 - 6.061) | 1.069 | (0.575 - 1.987) |
|  | >4.2 | 1.890 | (0.538 - 6.641) | 1.430 | (0.460 - 4.447) | 3.459 | (1.180 - 10.14) | 1.676 | (0.861 - 3.262) |
|  |  |  |  |  |  |  |  |  |  |
| Model 3 | <1.1 | Ref | -- | Ref | -- | Ref | -- | Ref | -- |
|  | 1.2-2.2 | 0.712 | (0.144 - 3.507) | 0.416 | (0.126 - 1.373) | 0.606 | (0.168 - 2.182) | 0.618 | (0.332 - 1.150) |
|  | 2.3-4.2 | 1.438 | (0.509 - 4.061) | 0.778 | (0.263 - 2.296) | 2.107 | (0.554 - 8.013) | 1.088 | (0.584 - 2.025) |
|  | >4.2 | 1.711 | (0.555 - 5.275) | 1.540 | (0.520 - 4.562) | 4.173 | (1.175 - 14.82) | 1.657 | (0.865 - 3.177) |
|  |  |  |  |  |  |  |  |  |  |
| Model 4 | <1.1 | Ref | -- | Ref | -- | Ref | -- | Ref | -- |
|  | 1.2-2.2 | 0.596 | (0.132 - 2.693) | 0.442 | (0.126 - 1.545) | 0.653 | (0.194 - 2.200) | 0.563 | (0.291 - 1.092) |
|  | 2.3-4.2 | 1.503 | (0.475 - 4.754) | 0.997 | (0.231 - 4.293) | 2.021 | (0.543 - 7.514) | 1.063 | (0.533 - 2.116) |
|  | >4.2 | 1.289 | (0.463 - 3.583) | 1.903 | (0.485 - 7.475) | 4.641* | (1.358 - 15.85) | 1.610 | (0.810 - 3.200) |
|  |  |  |  |  |  |  |  |  |  |
| Model 5 | <1.1 | Ref | -- | Ref | -- | Ref | -- | Ref | -- |
|  | 1.2-2.2 | 0.401 | (0.0690 - 2.329) | 0.424 | (0.0979 - 1.834) | 0.689 | (0.157 - 3.023) | 0.520 | (0.250 - 1.084) |
|  | 2.3-4.2 | 1.050 | (0.410 - 2.686) | 0.925 | (0.206 - 4.159) | 2.529 | (0.665 - 9.613) | 1.006 | (0.508 - 1.994) |
|  | >4.2 | 1.237 | (0.501 - 3.054) | 1.712 | (0.397 - 7.380) | 5.091* | (1.448 - 17.90) | 1.520 | (0.774 - 2.987) |
|  |  |  |  |  |  |  |  |  |  |
| Model 6 | <1.1 | -- | -- | Ref | -- | Ref | -- | -- | -- |
|  | 1.2-2.2 | -- | -- | 0.404 | (0.0860 - 1.895) | 0.636 | (0.124 - 3.260) | -- | -- |
|  | 2.3-4.2 | -- | -- | 0.892 | (0.161 - 4.948) | 2.681 | (0.690 - 10.41) | -- | -- |
|  | >4.2 | -- | -- | 2.027 | (0.367 - 11.18) | 5.727** | (1.744 - 18.80) | -- | -- |

* - p < 0.025 ; ** - p < 0.01

Model 1: adjusted for age, sex, and urinary creatinine concentration

Model 2: further adjusted for race/ethnicity, income, smoking, body mass index, and waist circumference

Model 3: veteran/military status, citizenship status, marital status, household size, pregnancy status, language at subject interview, health insurance coverage, and employment status in the prior week

Model 4: consumption of bottled water in the past 24 hrs, consumption of alcohol, and annual consumption of tuna fish

Model 5: presence of emotional support in one’s life, being on a diet, using a water treatment device, access to a routine source of health care, vaccinated for Hepatitis A or B, consumption of dietary supplements (vitamins or minerals), and inability to purchase balanced meals on a consistent basis

Model 6: concentration of (2-ethylhexyl) phthalate (MEHP), mono-isobutyl phthalate (MiBP), and mono-n-butyl phthalate (MeBP)
