# Supplementary material for: Disconcordance in Statistical Models of Bisphenol A and Chronic Disease Outcomes in NHANES 2003-08
Source: PLoS One. 2013 Nov 6;8(11):e79944. doi: 10.1371/journal.pone.0079944 (PMC3819299; doi:10.1371/journal.pone.0079944)
Supplement: Table S16 — Dose-response regression analysis of self-reported diabetes for NHANES 03-04 (N = 1,455), 05-06 (N = 1,498), 07-08 (N = 1,705), and a pooled sample (N = 4,658). (DOCX) [file pone.0079944.s016.docx]

Table S16. Dose-response regression analysis of self-reported diabetes for NHANES 03-04 (N = 1,455), 05-06 (N = 1,498), 07-08 (N = 1,705), and a pooled sample (N = 4,658).

|  |  | **NHANES 03-04** | | **NHANES 05-06** | | **NHANES 07-08** | | **Pooled** |  |
| --- | --- | --- | --- | --- | --- | --- | --- | --- | --- |
|  | **[BPA] (ng/ml)** | **OR (95% CI)** | | **OR (95% CI)** | | **OR (95% CI)** | | **OR (95% CI)** | |
| Model 1 | <1.1 | Ref | -- | Ref | -- | Ref | -- | Ref | -- |
|  | 1.2-2.2 | 1.653 | (0.992 - 2.755) | 2.048* | (1.109 - 3.781) | 1.374 | (0.838 - 2.251) | 1.682** | (1.252 - 2.258) |
|  | 2.3-4.2 | 1.575 | (0.969 - 2.560) | 1.705 | (0.663 - 4.381) | 1.489 | (0.730 - 3.039) | 1.593 | (1.036 - 2.451) |
|  | >4.2 | 3.081** | (2.020 - 4.701) | 2.407 | (1.026 - 5.645) | 0.920 | (0.480 - 1.762) | 1.940** | (1.332 - 2.824) |
|  |  |  |  |  |  |  |  |  |  |
| Model 2 | <1.1 | Ref | -- | Ref | -- | Ref | -- | Ref | -- |
|  | 1.2-2.2 | 1.634 | (0.751 - 3.554) | 1.880 | (1.053 - 3.355) | 1.389 | (0.867 - 2.225) | 1.614** | (1.144 - 2.275) |
|  | 2.3-4.2 | 1.679 | (0.887 - 3.179) | 1.568 | (0.588 - 4.178) | 1.581 | (0.889 - 2.811) | 1.493 | (0.947 - 2.355) |
|  | >4.2 | 2.933** | (1.750 - 4.916) | 2.236 | (0.749 - 6.671) | 0.867 | (0.459 - 1.638) | 1.806** | (1.170 - 2.787) |
|  |  |  |  |  |  |  |  |  |  |
| Model 3 | <1.1 | Ref | -- | Ref | -- | Ref | -- | Ref | -- |
|  | 1.2-2.2 | 1.642 | (0.728 - 3.706) | 1.870* | (1.103 - 3.169) | 1.317 | (0.799 - 2.170) | 1.632** | (1.147 - 2.321) |
|  | 2.3-4.2 | 1.722 | (0.870 - 3.408) | 1.613 | (0.701 - 3.712) | 1.524 | (0.854 - 2.717) | 1.529 | (0.989 - 2.363) |
|  | >4.2 | 2.822** | (1.638 - 4.862) | 2.531 | (0.886 - 7.226) | 0.837 | (0.408 - 1.718) | 1.812** | (1.170 - 2.805) |
|  |  |  |  |  |  |  |  |  |  |
| Model 4 | <1.1 | Ref | -- | Ref | -- | Ref | -- | Ref | -- |
|  | 1.2-2.2 | 1.517 | (0.685 - 3.361) | 2.001* | (1.111 - 3.603) | 1.200 | (0.699 - 2.058) | 1.610* | (1.122 - 2.311) |
|  | 2.3-4.2 | 1.653 | (0.875 - 3.124) | 1.605 | (0.689 - 3.738) | 1.307 | (0.723 - 2.363) | 1.476 | (0.958 - 2.276) |
|  | >4.2 | 2.524** | (1.468 - 4.339) | 2.902 | (0.951 - 8.851) | 0.778 | (0.372 - 1.630) | 1.793* | (1.147 - 2.801) |
|  |  |  |  |  |  |  |  |  |  |
| Model 5 | <1.1 | Ref | -- | Ref | -- | Ref | -- | Ref | -- |
|  | 1.2-2.2 | 1.282 | (0.520 - 3.162) | 1.742 | (0.984 - 3.085) | 1.092 | (0.594 - 2.007) | 1.443 | (0.982 - 2.119) |
|  | 2.3-4.2 | 1.637 | (0.809 - 3.312) | 1.556 | (0.668 - 3.624) | 1.323 | (0.715 - 2.446) | 1.512 | (0.998 - 2.289) |
|  | >4.2 | 2.401** | (1.405 - 4.103) | 2.816 | (1.030 - 7.699) | 0.755 | (0.371 - 1.539) | 1.760* | (1.137 - 2.724) |
|  |  |  |  |  |  |  |  |  |  |
| Model 6 | <1.1 | -- | -- | Ref | -- | Ref | -- | -- | -- |
|  | 1.2-2.2 | -- | -- | 1.739 | (0.983 - 3.074) | 1.110 | (0.604 - 2.042) | -- | -- |
|  | 2.3-4.2 | -- | -- | 1.551 | (0.661 - 3.637) | 1.469 | (0.805 - 2.681) | -- | -- |
|  | >4.2 | -- | -- | 2.810 | (1.035 - 7.629) | 0.737 | (0.323 - 1.683) | -- | -- |

* - p < 0.025 ; ** - p < 0.01

Model 1: adjusted for age, sex, and urinary creatinine concentration

Model 2: further adjusted for race/ethnicity, income, smoking, body mass index, and waist circumference

Model 3: veteran/military status, citizenship status, marital status, household size, pregnancy status, language at subject interview, health insurance coverage, and employment status in the prior week

Model 4: consumption of bottled water in the past 24 hrs, consumption of alcohol, and annual consumption of tuna fish

Model 5: presence of emotional support in one’s life, being on a diet, using a water treatment device, access to a routine source of health care, vaccinated for Hepatitis A or B, consumption of dietary supplements (vitamins or minerals), and inability to purchase balanced meals on a consistent basis

Model 6: concentration of (2-ethylhexyl) phthalate (MEHP), mono-isobutyl phthalate (MiBP), and mono-n-butyl phthalate (MeBP)
