# Supplementary material for: Disconcordance in Statistical Models of Bisphenol A and Chronic Disease Outcomes in NHANES 2003-08
Source: PLoS One. 2013 Nov 6;8(11):e79944. doi: 10.1371/journal.pone.0079944 (PMC3819299; doi:10.1371/journal.pone.0079944)
Supplement: Table S17 — Marginal effects for logistic regression model in the analysis of self-reported CHD. (DOCX) [file pone.0079944.s017.docx]

Table S17. Marginal effects for logistic regression model in the analysis of self-reported CHD.

|  | **NHANES 03-04** | | **NHANES 05-06** | | **NHANES 07-08** | | **Pooled** |  |
| --- | --- | --- | --- | --- | --- | --- | --- | --- |
|  | **OR (95% CI)** | | **OR (95% CI)** | | **OR (95% CI)** | | **OR (95% CI)** | |
| Model 1 | 0.0080* | (0.0010 - 0.0149) | 0.0036 | (-0.0006 - 0.0078) | 0.0015 | (-0.0029 - 0.0059) | 0.0031 | (0.00004 - 0.0062) |
| Model 2 | 0.0108* | (0.0021 - 0.0195) | 0.0051* | (0.0008 - 0.0093) | 0.0029 | (-0.0017 - 0.0074) | 0.004* | (0.0009 - 0.0071) |
| Model 3 | 0.0151** | (0.0045 - 0.0256) | 0.0064* | (0.0010 - 0.0118) | 0.0024 | (-0.0028 - 0.0077) | 0.0039* | (0.0006 - 0.0071) |
| Model 4 | 0.0174** | (0.0076 - 0.0272) | 0.0073** | (0.0021 - 0.0125) | 0.0028 | (-0.0026 - 0.0082) | 0.0035* | (0.0006 - 0.0064) |
| Model 5 | 0.0169** | (0.0064 - 0.0275) | 0.0067** | (0.0020 - 0.0115) | 0.0025 | (-0.0030 - 0.0080) | 0.0032 | (0.0004 - 0.006) |
| Model 6 | -- | -- | 0.0070** | (0.0021 - 0.0119) | 0.0026 | (-0.0028 - 0.0081) | -- | -- |

* - p < 0.025 ; ** - p < 0.01

Model 1: adjusted for age, sex, and urinary creatinine concentration

Model 2: further adjusted for race/ethnicity, income, smoking, body mass index, and waist circumference

Model 3: veteran/military status, citizenship status, marital status, household size, pregnancy status, language at subject interview, health insurance coverage, and employment status in the prior week

Model 4: consumption of bottled water in the past 24 hrs, consumption of alcohol, and annual consumption of tuna fish

Model 5: presence of emotional support in one’s life, being on a diet, using a water treatment device, access to a routine source of health care, vaccinated for Hepatitis A or B, consumption of dietary supplements (vitamins or minerals), and inability to purchase balanced meals on a consistent basis

Model 6: concentration of (2-ethylhexyl) phthalate (MEHP), mono-isobutyl phthalate (MiBP), and mono-n-butyl phthalate (MeBP)
