# Supplementary material for: Disconcordance in Statistical Models of Bisphenol A and Chronic Disease Outcomes in NHANES 2003-08
Source: PLoS One. 2013 Nov 6;8(11):e79944. doi: 10.1371/journal.pone.0079944 (PMC3819299; doi:10.1371/journal.pone.0079944)
Supplement: Table S18 — Marginal effects for logistic regression model in the analysis of self-reported diabetes. (DOCX) [file pone.0079944.s018.docx]

Table S18. Marginal effects for logistic regression model in the analysis of self-reported diabetes.

|  | **NHANES 03-04** | | **NHANES 05-06** | | **NHANES 07-08** | | **Pooled** |  |
| --- | --- | --- | --- | --- | --- | --- | --- | --- |
|  | **OR (95% CI)** | | **OR (95% CI)** | | **OR (95% CI)** | | **OR (95% CI)** | |
| Model 1 | 0.0225** | (0.0135 - 0.0315) | -0.0012 | (-0.0151 - 0.0127) | -0.0251 | (-0.0492 - -0.0010) | 0.0032 | (-0.00391 - 0.0103) |
| Model 2 | 0.0210** | (0.0141 - 0.0279) | -0.0018 | (-0.0217 - 0.0180) | -0.0233 | (-0.0457 - -0.0010) | 0.0049 | (-0.0013 - 0.0111) |
| Model 3 | 0.0190** | (0.0138 - 0.0243) | 0.0022 | (-0.0106 - 0.0150) | -0.0228 | (-0.0458 - 0.0001) | 0.0051 | (-0.0009 - 0.011) |
| Model 4 | 0.0186** | (0.0110 - 0.0262) | 0.0025 | (-0.0090 - 0.0139) | -0.0218 | (-0.0447 - 0.0011) | 0.0046 | (-0.0012 - 0.0104) |
| Model 5 | 0.0193** | (0.0094 - 0.0293) | 0.0005 | (-0.0088 - 0.0098) | -0.0202 | (-0.0402 - -0.0002) | 0.004 | (-0.0017 - 0.0097) |
| Model 6 | -- | -- | 0.0005 | (-0.0090 - 0.0099) | -0.0213 | (-0.0432 - 0.0005) | -- | -- |

* - p < 0.025 ; ** - p < 0.01

Model 1: adjusted for age, sex, and urinary creatinine concentration

Model 2: further adjusted for race/ethnicity, income, smoking, body mass index, and waist circumference

Model 3: veteran/military status, citizenship status, marital status, household size, pregnancy status, language at subject interview, health insurance coverage, and employment status in the prior week

Model 4: consumption of bottled water in the past 24 hrs, consumption of alcohol, and annual consumption of tuna fish

Model 5: presence of emotional support in one’s life, being on a diet, using a water treatment device, access to a routine source of health care, vaccinated for Hepatitis A or B, consumption of dietary supplements (vitamins or minerals), and inability to purchase balanced meals on a consistent basis

Model 6: concentration of (2-ethylhexyl) phthalate (MEHP), mono-isobutyl phthalate (MiBP), and mono-n-butyl phthalate (MeBP)
