# Supplementary material for: Disconcordance in Statistical Models of Bisphenol A and Chronic Disease Outcomes in NHANES 2003-08
Source: PLoS One. 2013 Nov 6;8(11):e79944. doi: 10.1371/journal.pone.0079944 (PMC3819299; doi:10.1371/journal.pone.0079944)
Supplement: Table S19 — Marginal effects for log-linear logistic regression model in the analysis of self-reported CHD. (DOCX) [file pone.0079944.s019.docx]

Table S19. Marginal effects for log-linear logistic regression model in the analysis of self-reported CHD.

|  | **NHANES 03-04** | | **NHANES 05-06** | | **NHANES 07-08** | | **Pooled** |  |
| --- | --- | --- | --- | --- | --- | --- | --- | --- |
|  | **OR (95% CI)** | | **OR (95% CI)** | | **OR (95% CI)** | | **OR (95% CI)** | |
| Model 1 | 0.0099 | (-0.0029 - 0.0226) | 0.0035 | (-0.0092 - 0.0162) | 0.0093 | (-0.0020 - 0.0205) | 0.0074 | (0.0006 - 0.0142) |
| Model 2 | 0.0144 | (-0.0003 - 0.0290) | 0.0036 | (-0.0081 - 0.0153) | 0.0102 | (0.0002 - 0.0203) | 0.0074 | (0.0008 - 0.0140) |
| Model 3 | 0.0168* | (0.0037 - 0.0299) | 0.0046 | (-0.0060 - 0.0152) | 0.0111 | (0.0011 - 0.0212) | 0.0073* | (0.0009 - 0.0137) |
| Model 4 | 0.0146** | (0.0054 - 0.0239) | 0.0060 | (-0.0062 - 0.0181) | 0.0112 | (0.0004 - 0.0221) | 0.0067 | (0.0002 - 0.0131) |
| Model 5 | 0.0132 | (0.0013 - 0.0251) | 0.0047 | (-0.0065 - 0.0158) | 0.0106 | (0.0004 - 0.0208) | 0.0061 | (-0.0001 - 0.0123) |
| Model 6 | -- | -- | 0.0103 | (-0.0020 - 0.0226) | 0.0109 | (0.0001 - 0.0216) | -- | -- |

* - p < 0.025 ; ** - p < 0.01

Model 1: adjusted for age, sex, and urinary creatinine concentration

Model 2: further adjusted for race/ethnicity, income, smoking, body mass index, and waist circumference

Model 3: veteran/military status, citizenship status, marital status, household size, pregnancy status, language at subject interview, health insurance coverage, and employment status in the prior week

Model 4: consumption of bottled water in the past 24 hrs, consumption of alcohol, and annual consumption of tuna fish

Model 5: presence of emotional support in one’s life, being on a diet, using a water treatment device, access to a routine source of health care, vaccinated for Hepatitis A or B, consumption of dietary supplements (vitamins or minerals), and inability to purchase balanced meals on a consistent basis

Model 6: concentration of (2-ethylhexyl) phthalate (MEHP), mono-isobutyl phthalate (MiBP), and mono-n-butyl phthalate (MeBP)
