# Supplementary material for: Disconcordance in Statistical Models of Bisphenol A and Chronic Disease Outcomes in NHANES 2003-08
Source: PLoS One. 2013 Nov 6;8(11):e79944. doi: 10.1371/journal.pone.0079944 (PMC3819299; doi:10.1371/journal.pone.0079944)
Supplement: Table S20 — Marginal effects for log-linear logistic regression model in the analysis of self-reported diabetes. (DOCX) [file pone.0079944.s020.docx]

Table S20. Marginal effects for log-linear logistic regression model in the analysis of self-reported diabetes.

|  | NHANES 03-04 | | NHANES 05-06 | | NHANES 07-08 | | Pooled |  |
| --- | --- | --- | --- | --- | --- | --- | --- | --- |
|  | OR (95% CI) | | OR (95% CI) | | OR (95% CI) | | OR (95% CI) | |
| Model 1 | 0.0297** | (0.0190 - 0.0403) | 0.0139 | (-0.0021 - 0.0298) | -0.0030 | (-0.0171 - 0.0110) | 0.0139** | (0.0052 - 0.0226) |
| Model 2 | 0.0273** | (0.0178 - 0.0368) | 0.0114 | (-0.0109 - 0.0336) | -0.0023 | (-0.0133 - 0.0087) | 0.0123* | (0.0029 - 0.0218) |
| Model 3 | 0.0263** | (0.0170 - 0.0355) | 0.0140 | (-0.0068 - 0.0349) | -0.0028 | (-0.0146 - 0.0091) | 0.0126** | (0.0035 - 0.0218) |
| Model 4 | 0.0236** | (0.0166 - 0.0306) | 0.0152 | (-0.0066 - 0.0371) | -0.0044 | (-0.0169 - 0.0081) | 0.0120** | (0.0030 - 0.0211) |
| Model 5 | 0.0232** | (0.0150 - 0.0314) | 0.0132 | (-0.0053 - 0.0317) | -0.0042 | (-0.0157 - 0.0072) | 0.0117** | (0.0034 - 0.0200) |
| Model 6 | -- | -- | 0.0123 | (-0.0064 - 0.0310) | -0.0084 | (-0.0216 - 0.0049) | -- | -- |

* - p < 0.025 ; ** - p < 0.01

Model 1: adjusted for age, sex, and urinary creatinine concentration

Model 2: further adjusted for race/ethnicity, income, smoking, body mass index, and waist circumference

Model 3: veteran/military status, citizenship status, marital status, household size, pregnancy status, language at subject interview, health insurance coverage, and employment status in the prior week

Model 4: consumption of bottled water in the past 24 hrs, consumption of alcohol, and annual consumption of tuna fish

Model 5: presence of emotional support in one’s life, being on a diet, using a water treatment device, access to a routine source of health care, vaccinated for Hepatitis A or B, consumption of dietary supplements (vitamins or minerals), and inability to purchase balanced meals on a consistent basis

Model 6: concentration of (2-ethylhexyl) phthalate (MEHP), mono-isobutyl phthalate (MiBP), and mono-n-butyl phthalate (MeBP)
