# Supplementary material for: Disconcordance in Statistical Models of Bisphenol A and Chronic Disease Outcomes in NHANES 2003-08
Source: PLoS One. 2013 Nov 6;8(11):e79944. doi: 10.1371/journal.pone.0079944 (PMC3819299; doi:10.1371/journal.pone.0079944)
Supplement: Table S21 — Marginal effects for dose-response regression model for the pooled data. (DOCX) [file pone.0079944.s021.docx]

Table S21. Marginal effects for dose-response regression model for the pooled data.

|  |  | **CHD** |  | **Diabetes** |  |
| --- | --- | --- | --- | --- | --- |
|  | **[BPA] (ng/ml)** | **OR (95% CI)** | | **OR (95% CI)** | |
| Model 1 | <1.1 | Ref | -- | Ref | -- |
|  | 1.2-2.2 | -0.0109 | (-0.0277 - 0.0059) | 0.0381** | (0.0175 - 0.0586) |
|  | 2.3-4.2 | 0.0030 | (-0.0130 - 0.0190) | 0.0341* | (0.0035 - 0.0647) |
|  | >4.2 | 0.0152 | (-0.0034 - 0.0338) | 0.0485** | (0.0219 - 0.0752) |
|  |  |  |  |  |  |
| Model 2 | <1.1 | Ref | -- | Ref | -- |
|  | 1.2-2.2 | -0.0127 | (-0.0288 - 0.0034) | 0.0327** | (0.0103 - 0.0552) |
|  | 2.3-4.2 | 0.0018 | (-0.0142 - 0.0177) | 0.0274 | (-0.0028 - 0.0576) |
|  | >4.2 | 0.0136 | (-0.0038 - 0.0309) | 0.0404** | (0.0116 - 0.0692) |
|  |  |  |  |  |  |
| Model 3 | <1.1 | Ref | -- | Ref | -- |
|  | 1.2-2.2 | -0.0125 | (-0.0285 - 0.0034) | 0.0331** | (0.0104 - 0.0559) |
|  | 2.3-4.2 | 0.0022 | (-0.0136 - 0.0180) | 0.0287* | (0.0001 - 0.0573) |
|  | >4.2 | 0.0131 | (-0.0036 - 0.0298) | 0.0402** | (0.0116 - 0.0688) |
|  |  |  |  |  |  |
| Model 4 | <1.1 | Ref | -- | Ref | -- |
|  | 1.2-2.2 | -0.0145* | (-0.0308 - 0.0018) | 0.0316** | (0.0090 - 0.0541) |
|  | 2.3-4.2 | 0.0015 | (-0.0154 - 0.0185) | 0.0258 | (-0.0018 - 0.0535) |
|  | >4.2 | 0.0120 | (-0.0052 - 0.0292) | 0.0387** | (0.0102 - 0.0672) |
|  |  |  |  |  |  |
| Model 5 | <1.1 | Ref | -- | Ref | -- |
|  | 1.2-2.2 | -0.0161* | (-0.0336 - 0.0014) | 0.0233* | (0.0001 - 0.0465) |
|  | 2.3-4.2 | 0.0002 | (-0.0162 - 0.0166) | 0.0263* | (0.0009 - 0.0516) |
|  | >4.2 | 0.0103 | (-0.0061 - 0.0268) | 0.0359** | (0.0094 - 0.0625) |

* - p < 0.025 ; ** - p < 0.01

Model 1: adjusted for age, sex, and urinary creatinine concentration

Model 2: further adjusted for race/ethnicity, income, smoking, body mass index, and waist circumference

Model 3: veteran/military status, citizenship status, marital status, household size, pregnancy status, language at subject interview, health insurance coverage, and employment status in the prior week

Model 4: consumption of bottled water in the past 24 hrs, consumption of alcohol, and annual consumption of tuna fish

Model 5: presence of emotional support in one’s life, being on a diet, using a water treatment device, access to a routine source of health care, vaccinated for Hepatitis A or B, consumption of dietary supplements (vitamins or minerals), and inability to purchase balanced meals on a consistent basis
