# Supplementary material for: Disconcordance in Statistical Models of Bisphenol A and Chronic Disease Outcomes in NHANES 2003-08
Source: PLoS One. 2013 Nov 6;8(11):e79944. doi: 10.1371/journal.pone.0079944 (PMC3819299; doi:10.1371/journal.pone.0079944)
Supplement: Table S22 — Main effect, with interacted coefficients by year, and marginal effects for CHD on Bisphenol A over various functional forms, in the pooled data using model 5 only (all covariates except for phthalates). Marginal effects are reported at the means of all covariates. (DOCX) [file pone.0079944.s022.docx]

Table S22. Main effect, with interacted coefficients by year, and marginal effects for CHD on Bisphenol A over various functional forms, in the pooled data using model 5 only (all covariates except for phthalates). Marginal effects are reported at the means of all covariates.

|  |  |  | **Linear** |  | **Log-linear** |  | **Dose-Response** | | | |
| --- | --- | --- | --- | --- | --- | --- | --- | --- | --- | --- |
|  |  |  | **standardized BPA** |  | **log(BPA)** |  | **Q1** | **Q2** | **Q3** | **Q4** |
| Main Effect | BPA | coef. | 0.527** |  | 0.494** |  | ref. | -0.26 | 0.561 | 0.518 |
|  |  | 95% CI | [0.280,0.773] |  | [0.151,0.837] |  | ref. | [-1.608,1.088] | [-0.105,1.227] | [-0.197,1.233] |
|  | 05-06*BPA | coef. | -0.455** |  | -0.488 |  | ref. | -0.6 | -1.139 | -0.407 |
|  |  | 95% CI | [-0.700,-0.211] |  | [-0.934,-0.0409] |  | ref. | [-2.520,1.320] | [-2.393,0.116] | [-1.654,0.841] |
|  | 07-08*BPA | coef. | -0.470* |  | -0.202 |  | ref. | -0.488 | -0.372 | 0.279 |
|  |  | 95% CI | [-0.844,-0.0955] |  | [-0.707,0.303] |  | ref. | [-2.193,1.217] | [-1.583,0.838] | [-1.033,1.590] |
|  |  |  |  |  |  |  |  |  |  |  |
| Marginal effects | 2003 | δCHD/δBPA | 0.00190** |  | 0.00181* |  | ref. | -0.00067 | 0.00219 | 0.00198 |
|  |  | 95% CI | [0.000569,0.00323] |  | [0.000206,0.00341] |  | ref. | [-0.00407,0.00273] | [-0.00113,0.00551] | [-0.00075,0.0047] |
|  | 2005 | δCHD/δBPA | 0.000459 |  | 0.0000417 |  | ref. | -0.00487 | -0.0037 | 0.00099 |
|  |  | 95% CI | [-0.000369,0.00129] |  | [-0.00245,0.00253] |  | ref. | [-0.01244,0.00271] | [-0.01091,0.00352] | [-0.0091,0.01107] |
|  | 2007 | δCHD/δBPA | 0.000315 |  | 0.00163 |  | ref. | -0.00256 | 0.00101 | 0.00588 |
|  |  | 95% CI | [-0.00110,0.00173] |  | [-0.00106,0.00433] |  | ref. | [-0.00626,0.00113] | [-0.00468,0.0067] | [-0.00377,0.01553] |

* - p < 0.025 ; ** - p < 0.01
