# Supplementary material for: Disconcordance in Statistical Models of Bisphenol A and Chronic Disease Outcomes in NHANES 2003-08
Source: PLoS One. 2013 Nov 6;8(11):e79944. doi: 10.1371/journal.pone.0079944 (PMC3819299; doi:10.1371/journal.pone.0079944)
Supplement: Table S23 — Main effect, with interacted coefficients by year, and marginal effects for Diabetes on Bisphenol A over various functional forms, in the pooled data using model 5 only (all covariates except for phthalates). Marginal effects are reported at the means of all covariates. (DOCX) [file pone.0079944.s023.docx]

Table S23. Main effect, with interacted coefficients by year, and marginal effects for Diabetes on Bisphenol A over various functional forms, in the pooled data using model 5 only (all covariates except for phthalates). Marginal effects are reported at the means of all covariates.

|  |  |  | **Linear** |  | **Log-linear** |  | **Dose-Response** | | | |
| --- | --- | --- | --- | --- | --- | --- | --- | --- | --- | --- |
|  |  |  | **standardized BPA** |  | **log(BPA)** |  | **Q1** | **Q2** | **Q3** | **Q4** |
| Main Effect | BPA | coef. | 0.412** |  | 0.340** |  | ref. | 0.399 | 0.426 | 0.813* |
|  |  | 95% CI | [0.175,0.649] |  | [0.154,0.525] |  | ref. | [-0.413,1.210] | [-0.198,1.050] | [0.172,1.453] |
|  | 05-06*BPA | coef. | -0.418** |  | -0.203 |  | ref. | 0.0568 | -0.199 | -0.0963 |
|  |  | 95% CI | [-0.694,-0.142] |  | [-0.560,0.154] |  | ref. | [-1.007,1.120] | [-1.281,0.882] | [-1.267,1.074] |
|  | 07-08*BPA | coef. | -0.678** |  | -0.266 |  | ref. | -0.133 | 0.148 | -0.679 |
|  |  | 95% CI | [-1.103,-0.254] |  | [-0.540,0.00856] |  | ref. | [-1.133,0.867] | [-0.708,1.004] | [-1.574,0.216] |
|  |  |  |  |  |  |  |  |  |  |  |
| Marginal effects | 2003 | δCHD/δBPA | 0.0129** |  | 0.0106** |  | ref. | 0.01 | 0.01083 | 0.02518** |
|  |  | 95% CI | [0.00507,0.0208] |  | [0.00473,0.0165] |  | ref. | [-0.01077,0.03077] | [-0.00436,0.02603] | [0.00671,0.04365] |
|  | 2005 | δCHD/δBPA | -0.000231 |  | 0.00509 |  | ref. | 0.01565 | 0.00697 | 0.02802 |
|  |  | 95% CI | [-0.00549,0.00503] |  | [-0.00594,0.0161] |  | ref. | [-0.00429,0.0356] | [-0.01797,0.03191] | [-0.01307,0.06911] |
|  | 2007 | δCHD/δBPA | -0.0103 |  | 0.00289 |  | ref. | 0.00942 | 0.02366 | 0.00445 |
|  |  | 95% CI | [-0.0238,0.00321] |  | [-0.00498,0.0108] |  | ref. | [-0.01239,0.03123] | [-0.004,0.05132] | [-0.01592,0.02482] |

* - p < 0.025 ; ** - p < 0.01
